# WNK2 variants associated with familial osteoarthritis alter the chondrocyte response to hyperosmotic stress

**DOI:** 10.1136/rmdopen-2025-005707

**Published:** 2025-07-01

**Authors:** Shivakumar R Veerabhadraiah, Derek J Matheson, Matthew Honeggar, Collin Aslor, Antonio C Zelada, Chris Stubben, Gregory J Stoddard, Nikolas H Kazmers, David J Grunwald, Michael J Jurynec

**Affiliations:** 1 Department of Orthopaedics, University of Utah Health, Salt Lake, Utah, USA; 2 Huntsman Cancer Institute Cancer Hospital, Salt Lake, Utah, USA; 3 Division of Epidemiology, University of Utah Health, Salt Lake, Utah, USA; 4 Department of Human Genetics, University of Utah Health, Salt Lake, Utah, USA; 5 Department of Biomedical Engineering, University of Utah, Salt Lake City, Utah, USA

**Keywords:** Chondrocytes, Osteoarthritis, Polymorphism, Genetic, Inflammation

## Abstract

**Objective:**

Chondrocytes of the synovial joint sense and respond to changes in osmolarity to maintain joint homeostasis. We hypothesised that an abnormal response to osmotic stress is a contributing factor to loss of joint homeostasis and the development of osteoarthritis (OA). Our goal was to identify whether genetic variants affecting the response to osmotic stress were associated with susceptibility to OA.

**Methods:**

Genomic analysis of independent families with dominant inheritance of OA revealed novel *WNK2* coding variants that segregated with occurrence of OA. WNK2 expression was examined by immunohistochemistry on normal and osteoarthritic tissue isolated from humans and mice. Wild type (WT) and variant WNK2 functions were analysed by overexpression effects in immortalised and primary human chondrocytes; loss-of-function effects were analysed in *WNK2* mutant cells. Transcriptomic analyses were used to identify genes and pathways dependent on WNK2 function and hyperosmotic stress.

**Results:**

We identified novel coding variants of *WNK2* associated with familial erosive hand OA and foot OA. *WNK2* is expressed in chondrocytes and its expression is highly elevated in end-stage human and mouse OA joints. When challenged by hyperosmotic stress, chondrocytes initiate a remodelling response, altering expression of both anabolic and catabolic genes and pathways. However, the combination of elevated WNK2 expression and hyperosmotic stress promotes a WNK2-dependent OA-associated transcriptional response that is exacerbated by expression of the OA-associated *WNK2* variants.

**Conclusions:**

Our data indicate elevated WNK2 signalling is associated with heightened susceptibility to OA. We hypothesise the synergistic effects of hyperosmotic stress and high WNK2 activity promote the development of OA.

**WHAT IS ALREADY KNOWN ON THIS TOPIC**

- The synovial joint is a hyperosmotic environment. Cells of the synovial joint sense and respond to changes in osmolarity to maintain joint homeostasis. The molecular mechanisms of how cells of the joint sense and respond to hyperosmotic stress is largely unknown. We hypothesised that an abnormal response to hyperosmotic stress is a contributing factor to loss of joint homeostasis and the development of osteoarthritis (OA).

**WHAT THIS STUDY ADDS**

- We discovered alleles of *WNK2* associated with familial OA, supporting the hypothesis that alterations in the response to osmotic stress are a new vulnerability factor for OA. WNK2 mediates the response of chondrocytes to hyperosmotic stress, and the combination of elevated WNK2 expression and hyperosmotic stress promotes an OA-associated transcriptional response. Expression of the *WNK2* variants in the absence of hyperosmotic stress is sufficient to promote expression of pathways associated with OA, a response that is further amplified under conditions of hyperosmotic stress.

**HOW THIS STUDY MIGHT AFFECT RESEARCH, PRACTICE OR POLICY**

- We propose that increased WNK2 activity in combination with chronic hyperosmotic stress may be a clinical marker of early stages of OA development. The modulation of this novel OA-associated pathway may provide an unexplored target for therapeutic intervention.

## Introduction

 Chondrocytes of articular cartilage are surrounded by a pericellular matrix (PCM) and embedded in a dense extracellular matrix (ECM).^1^ The high concentration of polyanionic glycosaminoglycan (GAG) sidechains on proteoglycans in the PCM/ECM generates a hyperosmotic environment in healthy cartilage (350–450 mOsm).^2^,^4^ Synovial fluid of the joint is also hyperosmotic (295–404 mOsm).^5^,^7^ A hyperosmotic environment is thought to maintain homeostasis of the joint through positive regulation of GAG synthesis^8 9^ and by increasing expression of a select subset of chondrogenic genes while suppressing those associated with hypertrophy.^10^,^15^ This effect is likely context and tissue dependent, as some studies have reported negative effects of a hyperosmotic environment on cultured chondrocytes including reduced cell viability and chondrogenic differentiation.^4 16 17^

Although the joint is generally characterised as a high osmolarity environment, cells of the synovial joint are constantly exposed to changing osmolarity due to the effects of everyday activity and injury, as well as physiological changes associated with ageing.^2^,^718^ The cells of the joint must continually respond to these changes, but the molecular and cellular pathways that directly sense and respond to changes in chronic osmotic stress over time are not well understood.^2 19 20^ In response to acute osmotic stress in vitro, chondrocytes undergo regulatory volume increase (RVI) or regulatory volume decrease (RVD) to maintain osmolarity.^2 19^ RVI and RVD are associated with changes in activity of ion channels that directly modulate the actin cytoskeleton and several intracellular signalling pathways, including ERK1/2, p38 and secondary messenger signalling.^221^,^31^ We hypothesised that altered response to osmotic stress may contribute to loss of homeostasis and the onset of osteoarthritis (OA). Identification of the genes and pathways that sense and respond to osmotic stress is important for understanding how the joint maintains homeostasis and investigating whether response to osmotic stress may facilitate the development of OA.

The With-No-Lysine (K) (WNK) protein kinases are intracellular sensors that respond to hyperosmotic stress by regulating ion channel activity and signalling pathways.^32^ In response to acute hyperosmotic stress, WNKs phosphorylate and activate the kinases SPAK and OSR1, which in turn phosphorylate and regulate sodium and potassium-chloride co-transporters to modulate osmoregulation.^33^ Though WNKs have not been directly associated with OA, ion channels targeted by WNKs are expressed in chondrocytes and upregulated in human OA tissue.^2 28 34^ *WNK2* is acutely downregulated in primary human chondrocytes in response to an OA-promoting stimulus in vitro,^35^ and WNK*2* is part of a gene expression module associated with knee OA susceptibility.^36^ These data suggest *WNK2* expression in chondrocytes may contribute to OA susceptibility, but its function has not been tested.

Here, we identify three novel coding variants in *WNK2* that segregated strictly with disease occurrence in families with inherited erosive hand OA (EHOA) and first metatarsophalangeal (MTP) joint OA. Consistent with the hypothesis that WNK2 modulates the response to physiological changes associated with OA, we find WNK2 is normally expressed in articular chondrocytes and is expressed at elevated levels in human and mouse tissues of osteoarthritic joints. We show that WNK2 function mediates a significant portion of the response to acute and chronic hyperosmotic stress in chondrocytes. Finally, we demonstrate that each of the OA-associated *WNK2* variants has increased function, driving a transcriptional response associated with promotion of OA. Our data provide strong support for the model that WNK2 functions in the chondrocyte response to hyperosmotic stress and that increased WNK2 signalling may drive the development of OA (online supplemental figure 1).

## Methods

Methods are included in the online supplemental material.

## Results

### Rare coding alleles of *WNK2* are associated with familial OA

We took an unbiased genetic approach to identify novel gene variants associated with familial increased risk of OA. Using a medical genetics database, the Utah Population Database,^37^,^40^ we identified 151 families in which OA appeared to be inherited as a dominant trait. We discovered three rare *WNK2* coding variants in four families diagnosed with bilateral EHOA or first MTP joint OA (table 1). Among analysed family members, *WNK2* variants were carried by all individuals with OA and absent from disease-free individuals. Each variant allele alters a region of the protein highly enriched in glutamines (Q-rich domains), which are necessary for phase separation and volume recovery in response to hyperosmotic stress in vitro.^41 42^ While all affected individuals have OA in the primary joint used for identification, family members often exhibited OA in additional joints, including knee, hip, shoulder and spine OA (online supplemental table 1). Our genetic studies indicate a striking correlation between inheritance of variants in *WNK2* and the occurrence of disease within families exhibiting OA in both weight-bearing and non-weight-bearing joints.

**Table 1:** *WNK2* variants identified in OA families

OA phenotype (family number)	Variant	Minor allele frequency	Protein domain affected by variant
1st MTP Joint OA (MTP24) and Erosive Hand OA (ERO32)	c.C2272A:p.H758N	0.0048	Q-rich domain
Erosive Hand OA (ERO549024)	c.G6161A:p.R2054Q	0.0000073	Q-rich domain
Erosive Hand OA (ERO20)	c.C3013T:p.L1005F	0.000013	Q-rich domain

OA, osteoarthritis.

### WNK2 expression is elevated in osteoarthritic tissue

**Figure 1:**
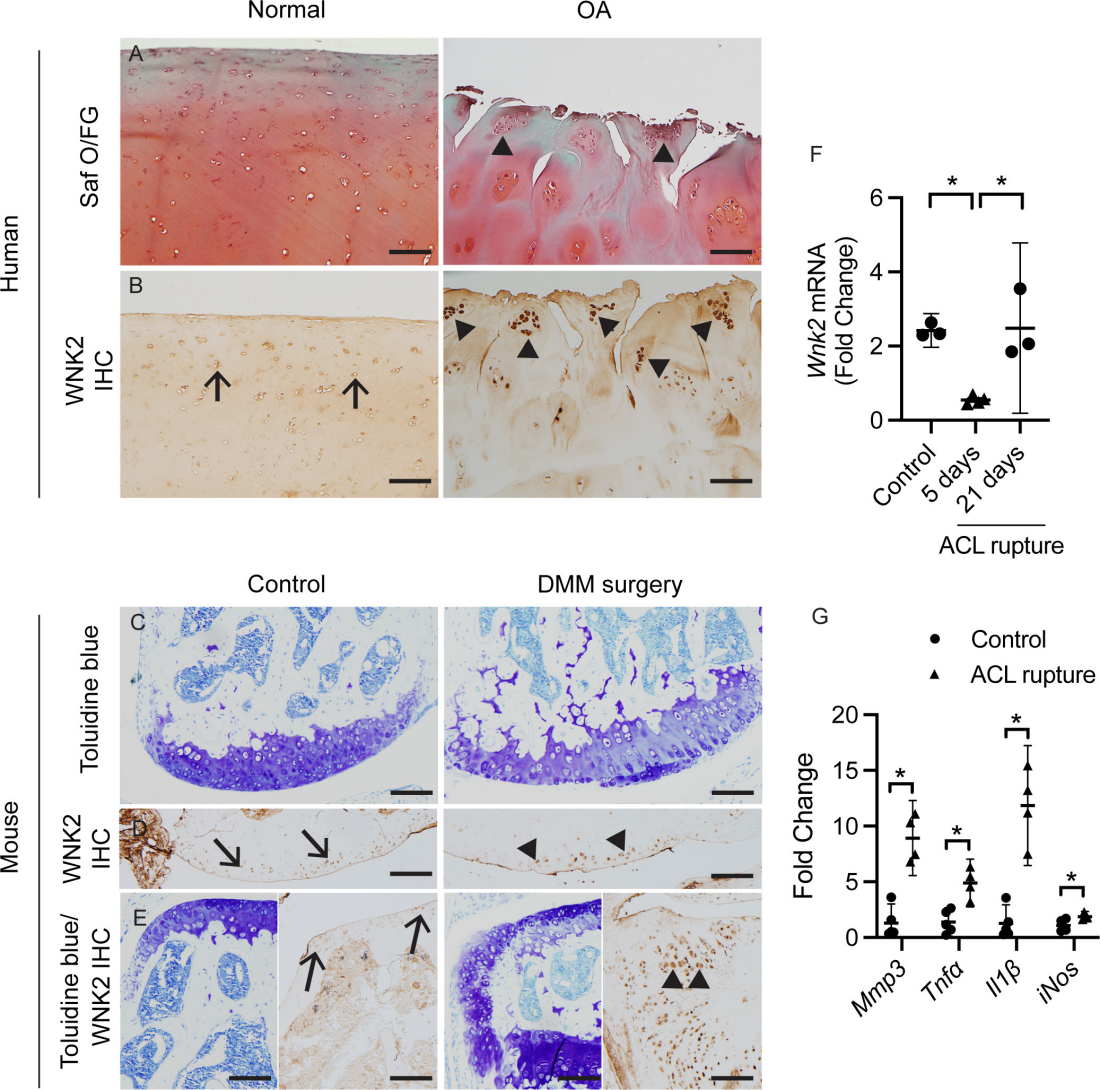
WNK2 expression is elevated in hypertrophic chondrocytes present in human osteoarthritic tissue and injured mouse knee joints. (**A**) Safranin O/Fast Green (Saf O/FG) stained human cartilage from the humeral head of healthy (Normal) and OA patients (OA). (**B**) Immunohistochemical staining demonstrates WNK2 expression in normal and osteoarthritic cartilage. WNK2 is detected at low levels in healthy human chondrocytes (**B**) and in the superficial layer of chondrocytes in uninjured mouse medial femoral condyle (**D**). Toluidine blue stained tissue sections (C—medial femoral condyle and E—osteophyte tissue) indicating loss of the superficial layer of articular cartilage after DMM surgery (**C**). In contrast, WNK2 is highly expressed in hypertrophic chondrocytes in damaged human OA cartilage (**B**) and in hypertrophic chondrocytes of the mouse medial femoral condyle 8 weeks post-DMM surgery (**C, D**). WNK2 is also highly expressed in osteophyte tissue 8 weeks post-DMM surgery (**E**). Arrows mark normal chondrocytes and arrowheads mark hypertrophic chondrocytes. Scale bar=100 µm. (**F**) *Wnk2* is acutely downregulated after anterior cruciate ligament (ACL) rupture (non-invasive injury) and restored 21 days post-ACL rupture. qPCR analysis using RNA isolated from whole control and ACL ruptured mouse knee joints 5 and 21days post-ACL rupture. Error bars represent mean with 95% CI and statistically significant differences of p≤0.05 (*) were determined by one-way ANOVA with Tukey’s multiple comparisons test, n=3. (**G**) OA-associated markers are upregulated in the mouse knee joint 5 days post-ACL rupture. Error bars represent mean with 95% CI and statistically significant differences of p≤0.05 (*) were determined by a two-tailed unpaired t-test, n=4. ANOVA, analysis of variance; DMM, destabilisation of the medial meniscus; IHC, immunohistochemistry; OA, osteoarthritis.

Given the association of *WNK2* variants and OA susceptibility, we characterised expression of WNK2 in normal and osteoarthritic joints (figure 1A–G and online supplemental figure 2). In normal human humeral head cartilage, WNK2 is present at low levels in chondrocytes (figure 1A,B). In contrast, it is strongly expressed in hypertrophic chondrocytes in damaged human osteoarthritic tissue (humeral head) harvested at the time of total shoulder arthroplasty (figure 1B). Similarly, WNK2 is expressed at modest levels in the superficial layer of articular chondrocytes in the uninjured mouse knee joint, but it is highly expressed in hypertrophic chondrocytes (figure 1C,D) and osteophyte tissue (figure 1E) 8 weeks after induction of OA by surgical destabilisation of the medial meniscus.^38^

### WNK2 expression is downregulated immediately after joint injury

*WNK2* is acutely downregulated in primary chondrocytes treated in vitro with fibronectin fragments^35^ and in human cartilage treated with IL1β (online supplemental table 2). We tested whether modulation of *Wnk2* expression was dynamically regulated after acute injury in mice in vivo. *Wnk2* mRNA was downregulated 5 days post-anterior cruciate ligament (ACL) rupture and recovers 21 days post-ACL rupture, despite the continued upregulation of inflammatory and catabolic genes (figure 1F,G).

### WNK2 mediates the acute response to hyperosmotic stress in chondrocytes

**Figure 2:**
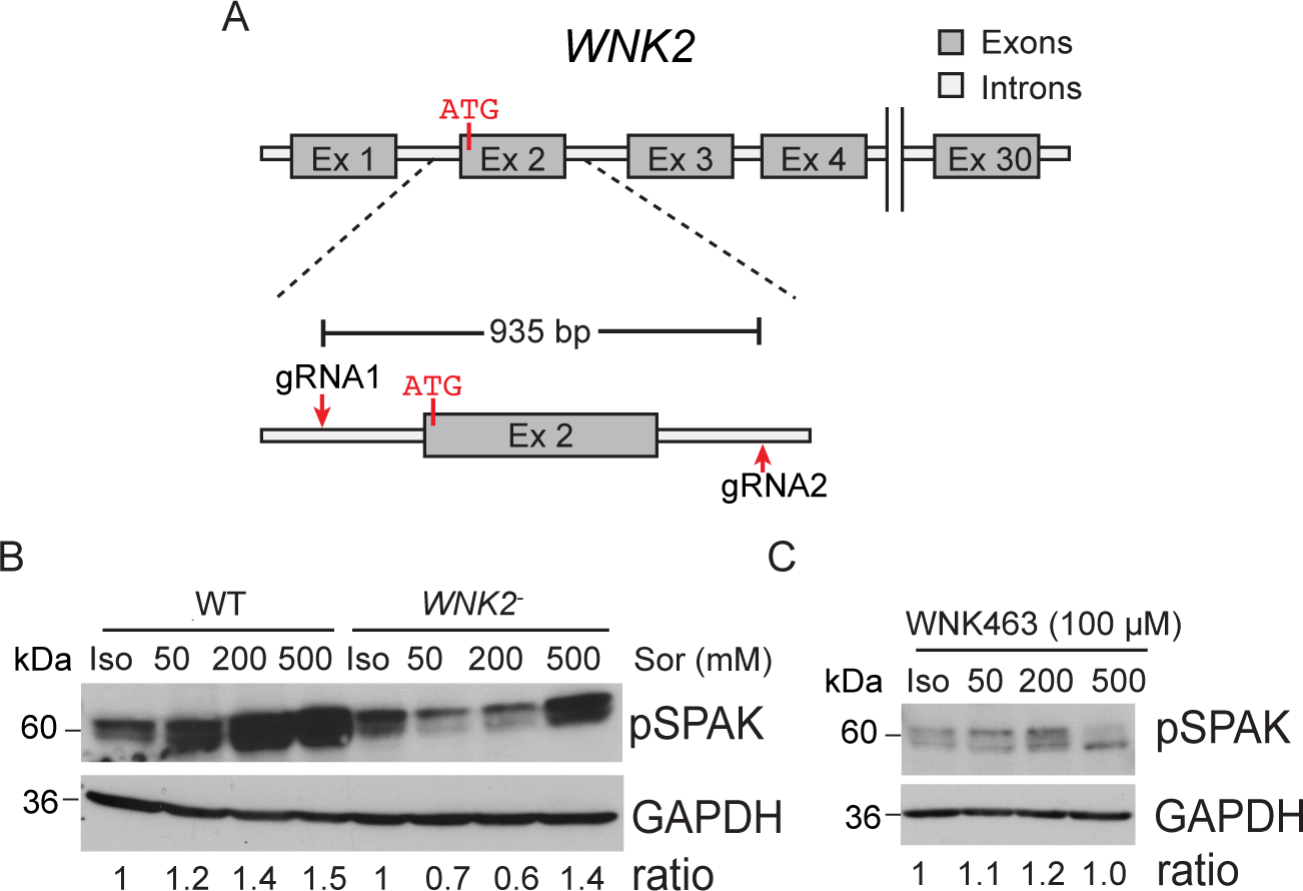
WNK2 mediates the acute response to hyperosmotic stress in chondrocytes. (**A**) Schematic representation of the *WNK2* locus and the guide RNAs (arrows) used to delete exon 2 of *WNK2* in T/C-28a2 chondrocytes using CRISPR/Cas9 genome editing. (**B**) *WNK2*^−^ chondrocytes do not respond to acute physiological levels of hyperosmotic stress. Immunoblot analysis indicating phosphorylated SPAK (pSPAK) protein levels in WT and *WNK2*^−^ chondrocytes in response to acute hyperosmotic stress. (**C**) pSPAK levels in WT chondrocytes exposed to hyperosmotic stress in the presence of a WNK1-4 inhibitor (WNK463). Ratio indicates the levels of pSPAK in each treatment relative to isotonic conditions. Glyceraldehyde 3-phosphate dehydrogenase (GAPDH) is used as a loading control. Iso, isotonic conditions; Sor, sorbitol concentration in cell culture media.

To determine the role of WNK2 in the response of chondrocytes to osmotic stress, we first generated T/C-28a2 human chondrocyte cells that lacked *WNK2* function to compare with the parental cells. We used CRISPR/Cas9 technology to delete exon 2, which includes the native start codon of *WNK2* (figure 2A and online supplemental figure 3).^43^ Analysis of RNA expression indicated only mutant transcripts lacking exon 2 sequences were generated in the WNK2 null cells (*WNK2*^−^) (online supplemental figure 3).

As an immediate response to acute hyperosmotic stress, WNK proteins directly phosphorylate the kinase effector proteins, SPAK and OSR1, which in turn modify ion channel functions to maintain osmotic balance.^44^ Similar to WNK2, SPAK and pSPAK are expressed in undamaged and damaged tissue from human OA joints (online supplemental figure 4). We examined the dose-dependent accumulation of phospho-SPAK (pSPAK) in WT or *WNK2^-^* chondrocytes exposed to acute hyperosmotic stress.^41^ Cells were treated with isotonic media (Iso/300 mOsm) or media supplemented with increasing levels of sorbitol (50 mM/350 mOsm, 200 mM/500 mOsm or 500 mM/800 mOsm) for 5 min, and protein extracts were collected for immunoblot analyses. Sorbitol was added to the medium to alter osmolarity^41^ as WNKs are inhibited by Cl^-^ ions.^45^ WT chondrocytes have a basal level of pSPAK (Iso) and respond to osmotic stress by increasing levels of pSPAK relative to isotonic conditions (figure 2B). In the presence of isotonic media, *WNK2*^−^ chondrocytes have a similar level of pSPAK compared with WT (figure 2B). However, when challenged with media containing 50 or 200 mM sorbitol, *WNK2^−^* chondrocytes fail to elevate pSPAK (figure 2B and online supplemental figure 5). The failure of *WNK2^−^* chondrocytes to elevate pSPAK is similar to chondrocytes exposed to hyperosmotic stress (50 or 200 mM) in the presence of a WNK1-4 inhibitor (WNK463, 100 µM)^46^ (figure 2C and online supplemental figure 5), indicating that WNK2 is largely responsible for SPAK phosphorylation at physiological levels of hyperosmotic stress. In contrast, both WT and *WNK2^−^* chondrocytes have high levels of pSPAK when exposed to supraphysiological levels of hyperosmotic stress (500 mM sorbitol), which is reduced by inhibiting WNK1-4 (figure 2C and online supplemental figure 5). Phosphorylation of SPAK in WT and *WNK2^−^* chondrocytes exposed to supraphysiological levels of hyperosmotic stress may be due to the presence of other WNKs, as all four *WNKs* are expressed at similar levels in WT and *WNK2^−^* chondrocytes (online supplemental table 3). Our data indicate that WNK2 activity is required for chondrocytes to mount the normal response to acute, physiological levels of hyperosmotic stress.

### Chronic hyperosmotic stress induces a remodelling response in chondrocytes

To determine the role of WNK2 under conditions of chronic hyperosmotic stress, we first determined if T/C-28a2 chondrocytes respond to chronic hyperosmotic stress. We performed targeted quantitative RT-PCR analysis using chondrocytes exposed to hyperosmotic stress (100 mM sorbitol/400 mOsm) for 7 days. Chronic hyperosmotic stress induced transcriptional changes similar to those observed in primary human chondrocytes treated with NaCl (online supplemental figure 6),^13^ including altered expression of OA-associated catabolic and anabolic markers (*MMP13*, *ADMATS1* and *SOX9*).

**Figure 3:**
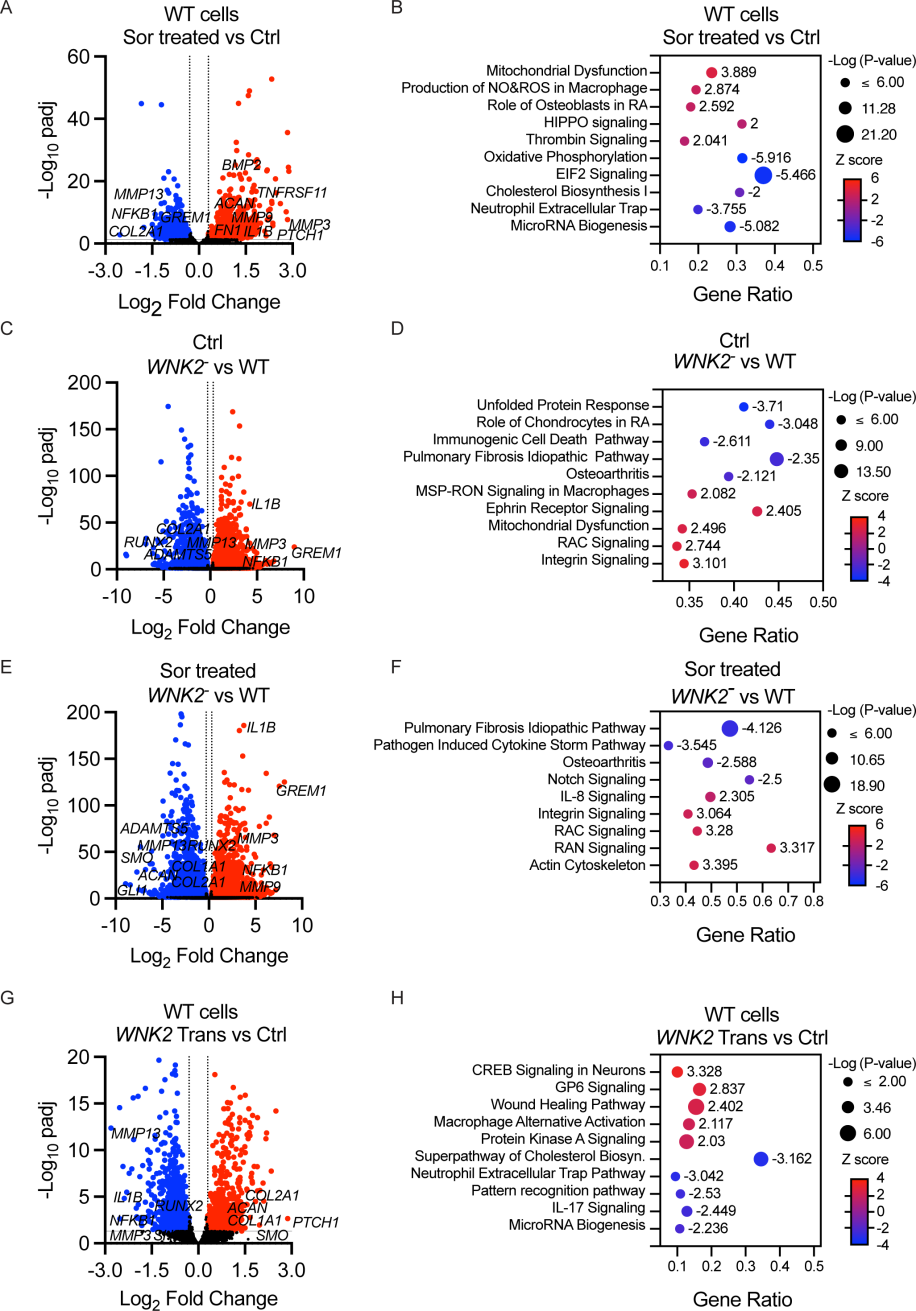
Chronic hyperosmotic stress or *WNK2* overexpression induces a remodelling response in chondrocytes, while loss of WNK2 function reduces OA-associated gene expression. (**A, C, E, G**) Comparative analysis of RNA-seq performed on RNA isolated from control or treated chondrocytes. The volcano plots indicate genes significantly upregulated (red) or downregulated (blue) in (**A**) WT chondrocytes treated for 7 days with 100 mM sorbitol (Sor) compared with control chondrocytes (Ctrl), (**C**) *WNK2*^−^ chondrocytes (*WNK2^-^*) compared with WT chondrocytes (WT) cultured in isotonic media, (**E**) *WNK2^−^* chondrocytes (*WNK2^−^*) compared with WT chondrocytes (WT) cultured in hyperosmotic media for 7 days (Sor treated), and (**G**) WT chondrocytes transfected with WT *WNK2* (*WNK2* Trans) as compared with control chondrocytes (Ctrl) cultured in isotonic media. (B, D, F, H) Bubble plots illustrate the top pathways identified from the differentially expressed genes in A, C, E, G using Ingenuity Pathway Analysis. In the bubble plots, the x-axis represents the gene ratio (number of genes in a pathway significantly altered by each treatment relative to the total number of genes in that pathway) while the y-axis displays the Ingenuity Pathway Analysis pathways. Each bubble represents a specific pathway, with the colour of the bubble indicating the positive (increased pathway gene expression, red) or negative (decrease pathway gene expression, blue) Z score. Darker colours indicate higher significance. The Z score is indicated next to each bubble. The size of the bubble indicates the −log (p value) and corresponds to the level of significant enrichment. n=4 for each condition. OA, osteoarthritis.

We performed RNA-seq analysis to determine the genome-wide transcriptional response of WT T/C-28a2 cells to hyperosmotic stress (online supplemental figure 7). Hyperosmotic stress resulted in upregulation of many anabolic genes (*ACAN*, *FGF2*, *BMP2* and *HIF1A*) and downregulation of catabolic genes known to have a role in OA pathogenesis (*MMP13, CXCL8* and *NFKB1*), although catabolic factors were also upregulated (*MMP3*, *MMP9* and *ADAMTS5*) (figure 3A). To discover pathways regulated by chronic hyperosmotic stress, we used Ingenuity Pathway Analysis (IPA) software to identify the top-ranking pathways determined by Z-score, which predicts the significance and directionality of pathway activation or inhibition. The response to chronic conditions of hyperosmotic stress is complex. Despite upregulation of some anabolic genes, chronic hyperosmotic stress also affected pathways linked to OA pathogenesis. The latter set of changes included upregulation of mitochondrial dysfunction, production of NO and ROS, thrombin signalling and a downregulation of oxidative phosphorylation, microRNA biogenesis and cholesterol biosynthesis (figure 3B), indicating that chondrocytes respond to chronic hyperosmotic stress by initiating a cellular remodelling response.

### WNK2 is necessary for OA-associated gene expression in chondrocytes exposed to chronic hyperosmotic stress

To identify the WNK2-dependent pathways that mediate the chondrocyte response to chronic hyperosmotic stress, we performed RNA-seq analysis of *WNK2*^−^ chondrocytes cultured in isotonic or chronic hyperosmotic conditions. In comparison with the parental WT cells, *WNK2^−^* chondrocytes cultured in isotonic media express lower levels of genes associated with OA pathogenesis (*MMP13, RUNX2*, *ADAMTS5* and *CXCL8*) (figure 3C). IPA analysis indicated the top pathways downregulated in *WNK2^−^* chondrocytes cultured in isotonic conditions are those associated with OA, the role of chondrocytes in rheumatoid arthritis signalling, pulmonary fibrosis (WNT signalling) and the unfolded protein response, while the top upregulated pathways are the RAC, Integrin and Ephrin signalling pathways (figure 3D). This pattern of gene expression is mostly unchanged in *WNK2*^−^ chondrocytes challenged with chronic hyperosmotic stress (figure 3E). Even when exposed to chronic hyperosmotic stress, *WNK2^−^* chondrocytes maintained lower expression of genes associated with OA, pulmonary fibrosis and pathogen-induced cytokine storm, while NOTCH, RAC, RAN and integrin signalling pathways were upregulated (figure 3F).

### *WNK2* overexpression induces a remodelling response in chondrocytes

Given loss of WNK2 function in chondrocytes reduces OA-associated gene expression (figure 3C–F) and *WNK2* is expressed at high levels in hypertrophic chondrocytes at advanced stages of OA in mouse and human OA tissue (figure 1B,C), we examined the transcriptional response of chondrocytes with elevated *WNK2*. We performed RNA-seq analysis on control chondrocytes or chondrocytes transfected with a plasmid encoding *WNK2*, both of which were cultured in isotonic conditions. *WNK2* overexpression caused transcriptional changes like those observed in chondrocytes exposed to chronic hyperosmotic stress, resulting in upregulation of anabolic genes (*ACAN*, *COL2A1*, *TGFB1* and *BMP2*) and downregulation of catabolic genes known to have a role in OA pathogenesis (*MMP9*, *GREM1* and *IL1B*) (figure 3A,G). *WNK2* overexpression resulted in the alteration of pathways that maintain chondrocyte homeostasis including the upregulation of CREB and PKA signalling, the alternative macrophage activation pathway, wound healing and downregulation of cholesterol biosynthesis, IL-17 signalling, and the pattern recognition receptor pathway (figure 3H). These data indicate that chondrocytes respond to chronic hyperosmotic stress or *WNK2* overexpression by regulating genes and pathways involved in cellular remodelling to maintain chondrocyte homeostasis. Chronic hyperosmotic stress had a greater effect on pro-OA pathways, while *WNK2* overexpression positively regulated more anabolic/homeostatic pathways.

### Elevated WNK2 and hyperosmotic stress interact to promote OA-associated gene expression

**Figure 4:**
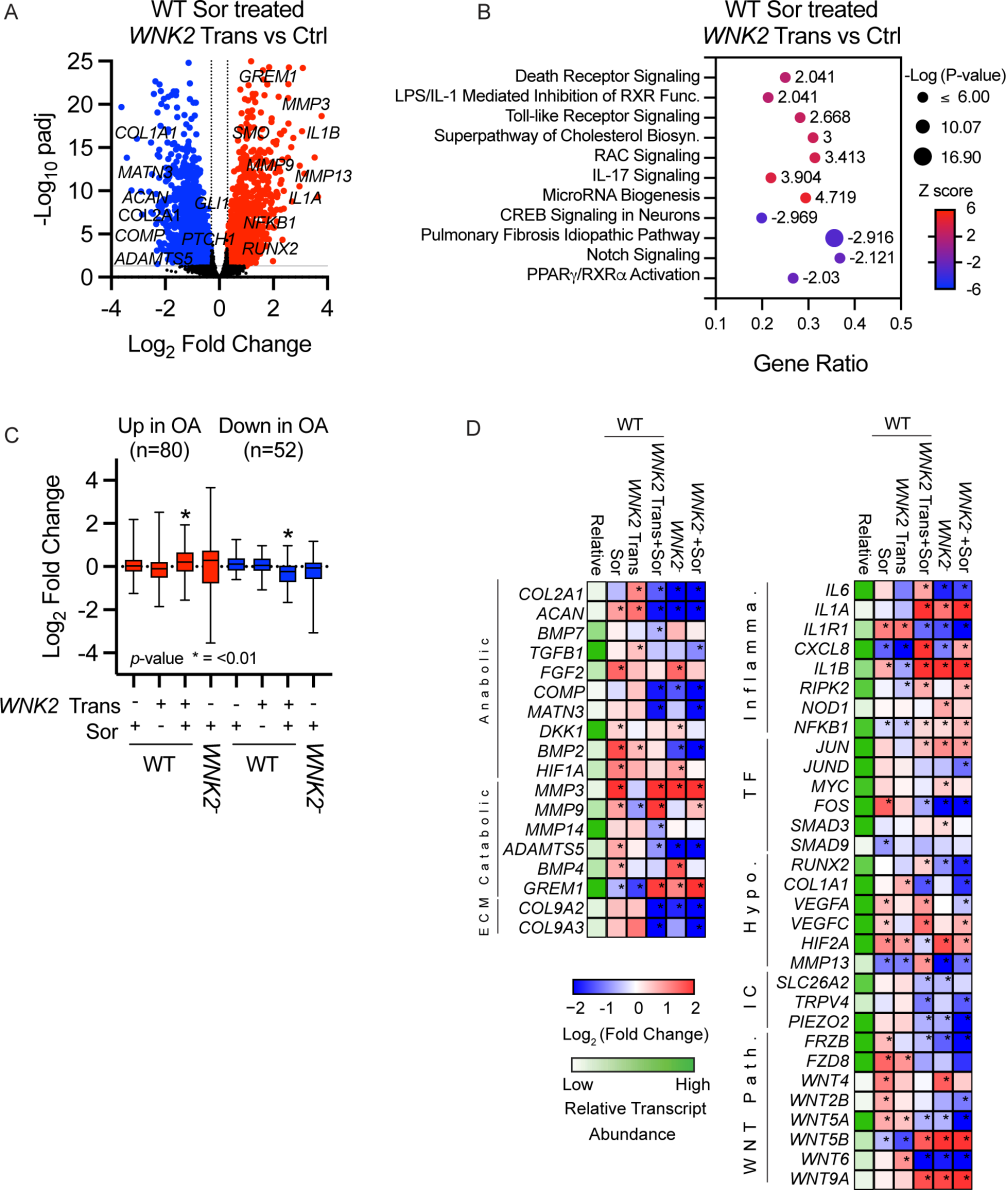
WNK2 acts synergistically with chronic hyperosmotic stress to induce expression of OA-associated genes and pathways. (**A, B**) Comparative analysis of RNA-seq performed on RNA isolated from WT or *WNK2*^−^ chondrocytes. (**A**) The volcano plot indicates genes significantly upregulated (red) or downregulated (blue) in WT chondrocytes transfected for 48 hours with *WNK2* and then treated for 7 days with 100 mM sorbitol (*WNK2* Trans) compared with control chondrocytes treated for 7 days with 100 mM (Ctrl). n=4 for each condition. (**B**) Bubble plot illustrates the top pathways identified from the differentially expressed genes in A. (**C**) We identified a common set of differentially expressed genes (DEGs) from five previously published human OA gene expression studies (see online supplemental methods); 82 upregulated and 52 downregulated DEGs shared between at least three studies were considered for comparison. The boxplot indicates log_2_ fold change for samples with and without *WNK2* transfection (*WNK2* Trans) and sorbitol treatment (Sor—100 mM) in WT and *WNK2^−^* chondrocytes. These genes were ranked by fold change and compared with a ranked list of fold changes for genes outside of the OA set (*Mann-Whitney p≤0.01). (**D**) Expression of selected OA-associated genes in WT and *WNK2^−^* transfected (Trans) or sorbitol (Sor) treated cells. Gene expression for all treatment groups was normalised to the respective counterpart with asterisks (*) indicating statistically significant differences of p<0.05. Blue tiles indicate repression of gene expression, and red tiles indicate induction of gene expression. Relative transcript abundance represents the mean of the normalised transcript counts of all samples. Green tiles indicate a high (>1000) relative transcript abundance and white tiles indicate a low (<10) relative transcript abundance. ECM, extra cellular matrix; Hypo, genes associated with hypertrophy; IC, ion channels; Inflamma., inflammation; OA, osteoarthritis; TF, transcription factor.

Elevated *WNK2* expression is a hallmark of the hypertrophic chondrocytes of OA tissue. We hypothesise that the increased *WNK2* expression over time interacts with continued hyperosmotic stress. We investigated the consequences of the combination of these factors. When chondrocytes overexpressing *WNK2* were exposed to chronic hyperosmotic stress, analysis of selected genes indicated the catabolic response was amplified as compared with untransfected cells (online supplemental figure 8). Thus, we examined the genome-wide effect on gene expression of chronic hyperosmotic stress on chondrocytes overexpressing *WNK2*. RNA-seq analysis indicated that in comparison with the parental line of T/C-28a2 cells, many catabolic genes were upregulated, including *MMP3*, *9*, and *13*, *IL1A*, *IL1Β*, *RUNX2*, *WNT5B*, *RIPK2* and *GREM1*, while many anabolic genes were downregulated, including *COL2A1*, *ACAN*, *COMP* and *MATN3* (figure 4A,D). IPA pathway analysis indicated that many pathways downregulated in response either to chronic hyperosmotic stress or *WNK2* overexpression were upregulated when chondrocytes overexpressing *WNK2* were exposed to hyperosmotic stress. Several upregulated pathways are involved in OA pathogenesis, including inflammation (IL-17, TLR, LPS/IL-1 pathways), cell death (death receptor signalling), RAC signalling and cholesterol biosynthesis. In contrast to hyperosmotic stress or increased WNK2 activity, CREB, NOTCH and PPARγ/RXRγ signalling pathways were downregulated in chondrocytes exposed to both hyperosmotic stress and WNK2 overexpression (figures3A,G  4B).

The combination of *WNK2* overexpression and chronic hyperosmotic stress in chondrocytes in vitro resulted in gene expression changes that might promote OA pathogenesis. We determined if these genes were similarly regulated in primary human chondrocytes. Indeed, primary human chondrocytes respond very similarly to hyperosmotic stress and *WNK2* overexpression as the expression of most genes was concordant with RNA-seq data from T/C-28a2 cells (online supplemental figure 9). Next, we asked how the chondrocyte response to hyperosmotic stress and *WNK2* overexpression related to gene expression in human OA tissue. We identified a core set of OA-associated genes from five previously published RNA-seq datasets (80 upregulated and 52 downregulated (online supplemental table 6).^3547^,^50^ Neither *WNK2* overexpression nor exposure to chronic hyperosmotic stress alone recapitulated the expression patterns observed in human OA tissue. In contrast, the gene expression pattern of chondrocytes subjected in vitro to chronic hyperosmotic stress and elevated WNK2 had a significant association with the pattern of expression observed in OA tissue (figure 4C). These results indicate that WNK2 and hyperosmotic stress act synergistically to induce transcriptional changes associated with OA, as neither overexpression of *WNK2* nor hyperosmotic stress alone could induce similar transcriptional responses.

We next wanted to determine whether WNK2 function and hyperosmotic stress contributed to additional chondrocyte gene pathways that are misregulated in OA. In addition to the response to osmotic stress, regulation of circadian clock expression is important in maintaining cartilage homeostasis. Disruption of the circadian clock gene expression is observed in human osteoarthritic cartilage,^51^,^53^ and Clock mutant mice develop accelerated spontaneous knee OA compared with WT.^54^ Furthermore, mechanical force and hyperosmotic stress interact to regulate the circadian clock in cartilage.^18^ We tested whether WNK2 activity might contribute to the regulation of clock genes in T/C-28a2 cells. We examined the expression of 17 key circadian clock genes in response to acute (2-hour sorbitol treatment) and chronic (7-day sorbitol treatment) hyperosmotic stress in our RNA-seq datasets from WT, *WNK2*^−^ and WT cells overexpressing *WNK2^R2054Q^* (online supplemental table 7).^55^ WT cells respond to acute hyperosmotic stress by altering the expression of many clock genes including *ARNTL*, *NPAS2*, *PER1*, *PER3* and *RORA*, while chronic hyperosmotic stress alters the expression of *ARNTL* and *NPAS2*. Compared with WT, *WNK2*^−^ cells have altered expression of several clock genes, including *CLOCK, NPAS2, CRY1, NR1D1, NRD1D2, RORA, DBP, TEF and USP2* under isotonic conditions, and several genes are altered in response to both acute and chronic hyperosmotic stress (online supplemental table 7). We next determined if overexpression of the OA-associated *WNK2^R2054Q^* variant in WT cells could alter the expression of clock genes. Under isotonic conditions, overexpression of *WNK2^R2054Q^* resulted in reduced expression of *NR1D1*, but in the presence of chronic hyperosmotic stress, many clock genes were altered, including *ARNTL*, *CLOCK*, *NPAS2*, *PER1*, *PER3*, *NR1D1*, *NR1D2*, *RORA*, *DBP*, *CIART* and *TEF* (online supplemental table 7). Finally, the expression of several clock genes in primary human chondrocytes was altered in response to *WNK2* overexpression (WT or *WNK2^R2054Q^*) in the presence of hyperosmotic stress (online supplemental figure 10). Together these data indicate that WNK2 activity is a regulator of clock gene expression in chondrocytes and that the *WNK2^R2054Q^* variant has an altered effect on clock gene expression compared with WT.

### *WNK2* variants associated with familial OA amplify the pro-OA transcriptional response to hyperosmotic stress

**Figure 5:**
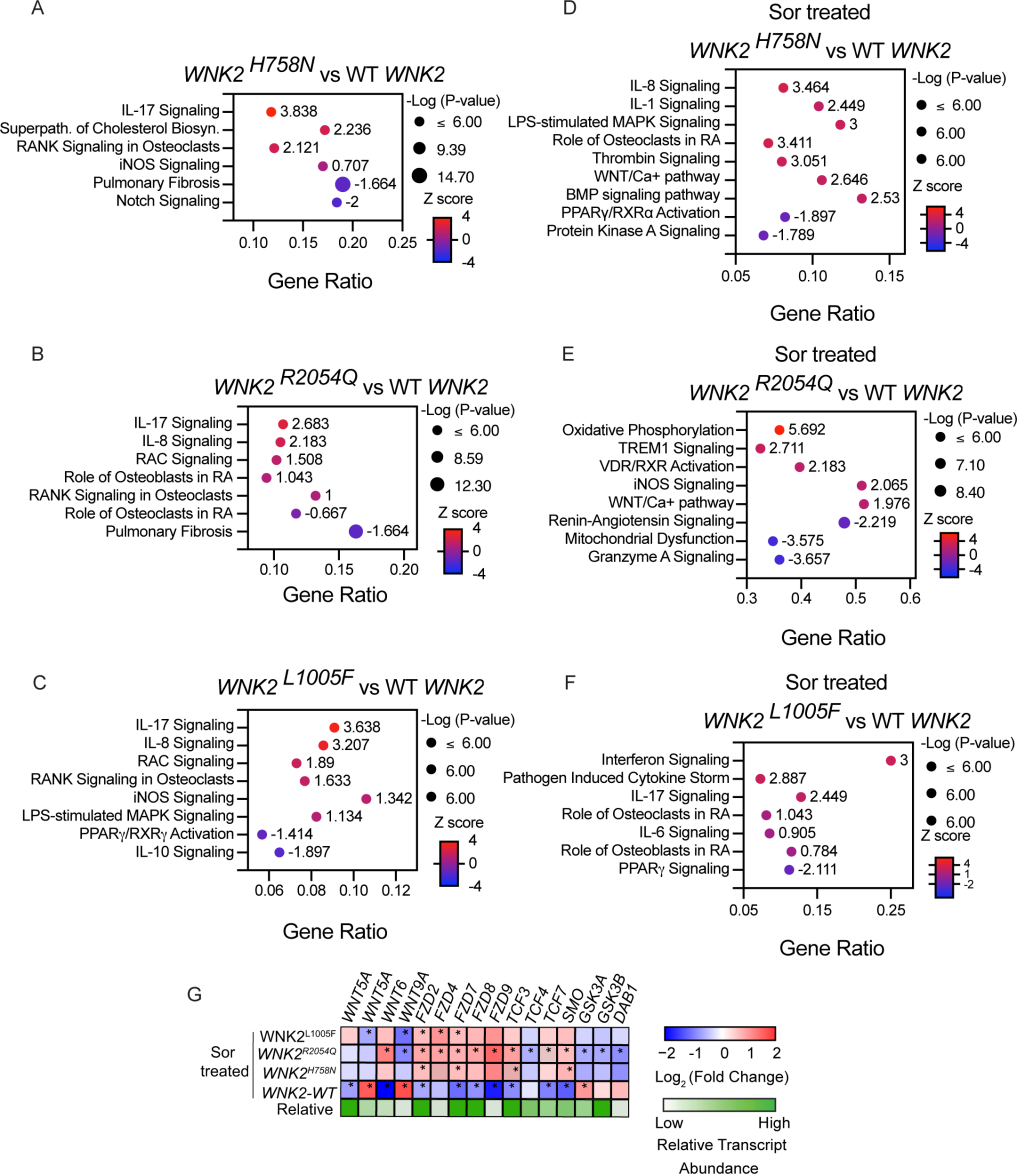
*WNK2* variants associated with familial OA amplify the pro-OA transcriptional response to hyperosmotic stress. Bubble plots illustrate the top pathways identified from the differentially expressed genes identified from RNA-seq performed on WT chondrocytes overexpressing WT *WNK2*, *WNK2^H758N^*, *WNK2^R2054Q^* or *WNK2^L1005F^* cultured in isotonic (**A–C**) or hyperosmotic media (**D–F**). (**G**) Expression of selected *WNT* pathway genes altered by *WNK2* variant overexpression and hyperosmotic stress. The WT *WNK2* overexpressing chondrocytes were compared with WT chondrocytes, and the *WNK2* variant overexpressing chondrocytes were compared with WT chondrocytes overexpressing WT *WNK2*. All cells were exposed to chronic hyperosmotic stress. Asterisks (*) indicate statistically significant differences in gene expression (p<0.05). n=4 for each condition. OA, osteoarthritis; LPS, lipopolysaccharide; RA, rheumatoid arthritis.

Having established effects of WNK2 expression and activity on gene expression in chondrocytes grown under isotonic or stressed conditions, we asked if the OA-associated *WNK2* variants might alter the effect on gene expression in response to isotonic or hyperosmotic conditions. To test this, the WT or each variant *WNK2* allele was overexpressed in chondrocytes and cultured in the absence of osmotic stress or exposed to chronic hyperosmotic stress (figure 5A–F). RNA-seq analysis indicated that simple overexpression of the OA-associated *WNK2* variants cultured in the absence of hyperosmotic stress promoted expression of pathways associated with OA (figure 5A–C), and a subset of OA-associated genes was also altered in response to *WNK2* overexpression (WT or *WNK2^R2054Q^*) in the presence of hyperosmotic stress in primary human chondrocytes (online supplemental figure 9). This includes upregulation of proinflammatory pathways (iNOS, IL-17, IL-8, IL-1 and IL-6), the cholesterol biosynthesis pathway, and RANK signalling pathways, while pathways that maintain joint homeostasis were downregulated (PPARγ, NOTCH and IL-10 signalling pathways) (figure 5A–C). This pro-OA gene expression response was further amplified when chondrocytes expressing the OA-associated *WNK2* variants were exposed to chronic hyperosmotic stress (figure 5D–F and online supplemental figures 7, 9 and 11), indicating the mutations as likely gain-of-function. For example, expression of *WNK2^H758N^* and *WNK2^L1005F^* variants in chondrocytes exposed to hyperosmotic stress led to further upregulation of IL-1, IL-8, IL-17, LPS signalling and downregulation of PPARγ signalling (figure 5A,C,D and F). WNT signalling has a known role in OA pathogenesis,^56^ and WNK activity regulates WNT signalling.^42 57 58^ In contrast to the effect of *WNK2* variant expression in isotonic conditions, when the variants are combined with chronic hyperosmotic stress, many genes in the WNT pathway were upregulated (figure 5E,G). In sum, these data indicate that all three WNK2 variants have a significant functional impact on WNK2 activity and that this altered WNK2 activity is sufficient to induce OA-associated gene expression in the absence of hyperosmotic stress.

## Discussion

The synovial joint contains a hyperosmotic environment that is subjected to modulation. Changes in osmolarity occur during mechanical loading, breakdown and synthesis of ECM, and pathological changes associated with ageing and OA.^2^,^718^ Cells are necessarily stressed in this dynamic environment, but the proteins that mediate responses to osmotic changes in the joint are largely unknown.^2 19 20^ We propose that under normal physiological conditions, chondrocytes use WNK2 to sense and mediate the response to hyperosmotic stress to maintain joint homeostasis, including the response to normal mechanical loading and everyday damage. We showed that when WT chondrocytes are exposed to increasing osmotic stress, they respond by elevating WNK2 activity. Our data indicate that chronic exposure to hyperosmotic stress or overexpression of *WNK2* in chondrocytes under isotonic conditions results in gene expression changes indicative of a remodelling response (eg, expression of both catabolic and anabolic markers) (figure 3). These results are consistent with previous studies demonstrating an anabolic response of chondrocytes exposed to hyperosmotic media.^8^,^1519 22^ However, our genome-wide transcriptional studies indicate the chondrocyte response to chronic osmotic stress is more complex than simply an anabolic response.

We have discovered that *WNK2* variants are associated with the recurrent onset of hereditary OA. We sought the origin of how WNK2 activity might contribute to the OA phenotype. We found WNK2 is highly expressed in damaged human OA cartilage and in hypertrophic chondrocytes in a mouse model of OA (figure 1). However, neither stress nor overexpression of WNK2 in vitro alone was sufficient to recapitulate the OA-like gene expression pattern. Remarkably, the combination of *WNK2* overexpression and chronic hyperosmotic stress has a distinct effect on gene expression. The two factors act synergistically to induce transcriptional changes associated with OA in vitro. As elevated WNK2 expression combined with chronic hyperosmotic stress is a characteristic of advanced OA tissue, we compared gene expression profiles of our in vitro model and OA tissue. The transcriptional profile of cultured chondrocytes with elevated WNK2 expression combined with chronic hyperosmotic stress overlaps significantly with genes expressed in human OA samples (figure 4).^3547^,^50^ We propose that the interaction between hyperosmotic stress and elevated WNK2 activity is an important driver of the OA phenotype. During disease progression, this interaction drives a catabolic response that degrades the ECM, leading to loss of GAGs and reduction in joint osmolarity, which is observed in end-stage OA tissues.^2^,^718^

Loss of WNK2 under normal conditions suppresses expression of OA-associated genes. Chondrocytes lacking *WNK2* have a significant downregulation of pathways associated with OA, rheumatoid arthritis, immunogenic cell death and fibrosis, and this response is not abrogated by hyperosmotic treatment (figure 3). Although pathway analysis suggests that *WNK2^-^* chondrocytes regulate some pathways that may be protective against the development of OA, they also have decreased expression of some anabolic markers (*COL2A1*, *ACAN* and *BMP2*) and upregulation of markers (*MMP3*, *MMP9*, *GREM1*, *IL1A*, *IL1B* and *RIPK2*) and pathways (mitochondrial dysfunction and integrin signalling) that are associated with the OA phenotype. These data suggest that a proper balance of WNK2 activity and hyperosmotic conditions is necessary to maintain joint homeostasis.

WNK2 is downregulated in the early stages of injury-induced OA in the mouse and in an in vitro model of OA,^35^ yet it is upregulated in advanced stages of OA (figure 1). The initial downregulation of WNK2 may be a protective mechanism to maintain joint homeostasis in response to acute injury (figures1  3). Over time, WNK2 is upregulated in the joint, and it responds to changes in osmotically stressed environments by upregulating an OA-associated transcriptional response, which disrupts joint homeostasis. It is known that healthy and OA joint tissues respond differentially to hyperosmotic stress,^6 10^ but the genes and pathways associated with this differential response remain unresolved. Changes in WNK2 expression during OA progression may partially mediate this response.

We identified several pathways in chondrocytes that were oppositely regulated by hyperosmotic stress or *WNK2* overexpression compared with the combination of *WNK2* overexpression with hyperosmotic stress. The cholesterol biosynthetic pathway and several inflammatory pathways were downregulated in response to hyperosmotic stress or *WNK2* overexpression but upregulated in the combined treatment. Mouse studies have demonstrated molecular pathways linking increased cholesterol metabolism to accelerated OA development.^59 60^ Further, inflammatory signalling pathways have emerged as key risk factors for OA susceptibility in humans and animal models.^38 39 61 62^ Our data suggest that normal WNK2 activity and hyperosmotic stress are necessary for the repression of the cholesterol biosynthetic and several inflammatory pathways (eg, IL-17 and pattern recognition receptor pathways). The upregulation of WNK2 during OA may cause coordinated expression of these pathways to disrupt joint homeostasis, leading to accelerated progression of OA.

From the in vitro studies with WT WNK2 overexpression combined with hyperosmotic stress, we anticipate the WNK2 variants would have elevated activity and promote the expression of OA-associated pathways. Indeed, functional analysis of the three OA-associated WNK2 variants demonstrated they are sufficient to induce OA-like gene expression even in the absence of hyperosmotic stress. Furthermore, this response is amplified when chondrocytes expressing the WNK2 variants are challenged with chronic hyperosmotic stress (figure 5 and online supplemental figures 7,9 and 10). Although these data suggest the *WNK2* variants are gain-of-function, further in vivo studies are needed to determine how they affect the acute and chronic response to hyperosmotic conditions. All three variants affect highly conserved amino acids in regions enriched in glutamines (Q-rich domains). The Q-rich domains are necessary for phase separation and volume recovery in vitro,^41^ and the C-terminus (where the *WNK2*^*R2054Q*^ variant is located) is required for Wnk activity in Drosophila.^42^ Previous work demonstrated that WNKs regulate WNT signalling.^42 57 63^ WNT signalling has an established role in OA pathogenesis, and inhibition of the pathway has been the focus of recent clinical trials.^64 65^ Our data suggest that WNK2 activity and chronic hyperosmotic stress may be an important modulator of WNT signalling and the onset or progression of OA.

Mechanotransduction,^66 67^ circadian clock gene expression^18 68^ and the response to osmotic stress^48^,^17^ are important pathways in maintaining cartilage homeostasis. There is an emerging link between these three biological factors. For example, the mechanosensitive ion channel, TRPV4, is also regulated by osmolarity,^23 30 31^ and Dudek *et al* demonstrated that mechanical force and hyperosmotic stress interact to regulate the circadian clock in cartilage.^18^ Finally, a recent OA genome-wide association study identified several loci containing genes that modulate the circadian clock.^69^ Our data indicate that WNK2 activity is a regulator of clock gene expression in chondrocytes and that the OA-associated *WNK2^R2054Q^* has an altered effect on clock gene expression compared with WT (online supplemental table 7 and online supplemental figure 10). It is possible that WNK2 is a critical osmotic-sensor and potentially mechano-sensor that regulates the circadian response in the joint. Future in vivo studies will allow us to examine genetic and physical interactions between WNK2 and key members of mechanotransduction and circadian rhythm pathways.

The study has several limitations. Our functional studies were performed in T/C-28a2 human chondrocytes, which is an immortalised costal chondrocyte cell line. Although we demonstrated that a subset of genes was also altered in response to *WNK2* overexpression (WT or *WNK2^R2054Q^*) in the presence of hyperosmotic stress in primary human chondrocytes (online supplemental figures 9 and 10), we are cautious not to overinterpret our data. Nevertheless, they demonstrate that novel coding variants in *WNK2* associated with familial EHOA and foot OA alter the biological function of WNK2. Whereas we focus on WNK2 in chondrocytes, we appreciate that WNK2 may have roles in other cells of the synovial joint. Generation of mice harbouring human OA alleles will allow us to determine the precise role of WNK2 in joint homeostasis and OA development.^38^

In sum, we propose that WNK2 is a major hyperosmotic sensor in chondrocytes that maintains joint homeostasis and that WNK2 activity, in combination with other factors such as chronic hyperosmotic stress, has a determinative role in the development of OA. We identified three novel coding variants in WNK2 that alter its function and are associated with familial OA. We found that WNK2 and chronic hyperosmotic treatment act synergistically to induce transcriptional changes resembling those observed in OA. A dysregulation of genes associated with cholesterol biosynthesis, inflammation, WNT signalling, the circadian rhythm and other cellular processes may contribute to OA pathogenesis through WNK2 and osmotic stress. Generation and analysis of loss-of-function and gain-of-function alleles of *Wnk2* will allow us to define the role of WNK2 in vivo in the context of the synovial joint and test if modulation of WNK2 activity is a potential therapeutic target.

## Supplementary material

10.1136/rmdopen-2025-005707 online supplemental file 1

## Data Availability

Data are available in a public, open access repository.

## References

[1] Wilusz RE, Sanchez-Adams J, Guilak F (2014). The structure and function of the pericellular matrix of articular cartilage. Matrix Biol.

[2] Lewis R, Feetham CH, Barrett-Jolley R (2011). Cell volume regulation in chondrocytes. Cell Physiol Biochem.

[3] Urban JP (1994). The chondrocyte: a cell under pressure. Br J Rheumatol.

[4] Urban JP, Hall AC, Gehl KA (1993). Regulation of matrix synthesis rates by the ionic and osmotic environment of articular chondrocytes. J Cell Physiol.

[5] Shanfield S, Campbell P, Baumgarten M (1988). Synovial fluid osmolality in osteoarthritis and rheumatoid arthritis. Clin Orthop Relat Res.

[6] Bertram KL, Krawetz RJ (2012). Osmolarity regulates chondrogenic differentiation potential of synovial fluid derived mesenchymal progenitor cells. Biochem Biophys Res Commun.

[7] Newman PJ, Grana WA (1988). The changes in human synovial fluid osmolality associated with traumatic or mechanical abnormalities of the knee. Arthroscopy.

[8] Xu X, Urban JPG, Tirlapur UK (2010). Osmolarity effects on bovine articular chondrocytes during three-dimensional culture in alginate beads. Osteoarthritis Cartilage.

[9] Negoro K, Kobayashi S, Takeno K (2008). Effect of osmolarity on glycosaminoglycan production and cell metabolism of articular chondrocyte under three-dimensional culture system. Clin Exp Rheumatol.

[10] Tew SR, Peffers MJ, McKay TR (2009). Hyperosmolarity regulates SOX9 mRNA posttranscriptionally in human articular chondrocytes. *Am J Physiol Cell Physiol*.

[11] Erndt-Marino J, Trinkle E, Hahn MS (2019). Hyperosmolar Potassium (K^+^) Treatment Suppresses Osteoarthritic Chondrocyte Catabolic and Inflammatory Protein Production in a 3-Dimensional In Vitro Model. Cartilage.

[12] Mongkhon JM, Thach M, Shi Q (2014). Sorbitol-modified hyaluronic acid reduces oxidative stress, apoptosis and mediators of inflammation and catabolism in human osteoarthritic chondrocytes. Inflamm Res.

[13] van der Windt AE, Haak E, Das RHJ (2010). Physiological tonicity improves human chondrogenic marker expression through nuclear factor of activated T-cells 5 in vitro. Arthritis Res Ther.

[14] Mang T, Lindemann S, Gigout A (2020). Increasing the Medium Osmolarity Reduces the Inflammatory Status of Human OA Chondrocytes and Increases Their Responsiveness to GDF-5. Int J Mol Sci.

[15] Govindaraj K, Meteling M, van Rooij J (2024). Osmolarity-Induced Altered Intracellular Molecular Crowding Drives Osteoarthritis Pathology. Adv Sci (Weinh).

[16] Jurgens WJFM, Lu Z, Zandieh-Doulabi B (2012). Hyperosmolarity and hypoxia induce chondrogenesis of adipose-derived stem cells in a collagen type 2 hydrogel. J Tissue Eng Regen Med.

[17] Parameswaran R, Kachroo U, Amirtham SM (2021). An in vitro analysis of the effect of hyperosmolarity on the chondrogenic potential of human articular cartilage derived chondroprogenitors. Tissue Cell.

[18] Dudek M, Pathiranage DRJ, Bano-Otalora B (2023). Mechanical loading and hyperosmolarity as a daily resetting cue for skeletal circadian clocks. Nat Commun.

[19] Hall AC (2019). The Role of Chondrocyte Morphology and Volume in Controlling Phenotype-Implications for Osteoarthritis, Cartilage Repair, and Cartilage Engineering. Curr Rheumatol Rep.

[20] Wilkins RJ, Browning JA, Urban JP (2000). Chondrocyte regulation by mechanical load. Biorheology.

[21] Kerrigan MJP, Hall AC (2008). Control of chondrocyte regulatory volume decrease (RVD) by [Ca2+]i and cell shape. Osteoarthritis Cartilage.

[22] Wang Z, Irianto J, Kazun S (2015). The rate of hypo-osmotic challenge influences regulatory volume decrease (RVD) and mechanical properties of articular chondrocytes. Osteoarthritis Cartilage.

[23] Fu S, Meng H, Inamdar S (2021). Activation of TRPV4 by mechanical, osmotic or pharmaceutical stimulation is anti-inflammatory blocking IL-1β mediated articular cartilage matrix destruction. Osteoarthritis Cartilage.

[24] Kerrigan MJP, Hook CSV, Qusous A (2006). Regulatory volume increase (RVI) by in situ and isolated bovine articular chondrocytes. J Cell Physiol.

[25] Erickson GR, Northrup DL, Guilak F (2003). Hypo-osmotic stress induces calcium-dependent actin reorganization in articular chondrocytes. Osteoarthritis Cartilage.

[26] Kurita T, Yamamura H, Suzuki Y (2015). The ClC-7 Chloride Channel Is Downregulated by Hypoosmotic Stress in Human Chondrocytes. Mol Pharmacol.

[27] Sánchez JC, López-Zapata DF (2010). Effects of osmotic challenges on membrane potential in human articular chondrocytes from healthy and osteoarthritic cartilage. Biorheology.

[28] Barrett-Jolley R, Lewis R, Fallman R (2010). The emerging chondrocyte channelome. Front Physiol.

[29] Erickson GR, Alexopoulos LG, Guilak F (2001). Hyper-osmotic stress induces volume change and calcium transients in chondrocytes by transmembrane, phospholipid, and G-protein pathways. J Biomech.

[30] Hdud IM, Mobasheri A, Loughna PT (2014). Effect of osmotic stress on the expression of TRPV4 and BKCa channels and possible interaction with ERK1/2 and p38 in cultured equine chondrocytes. *Am J Physiol Cell Physiol*.

[31] Phan MN, Leddy HA, Votta BJ (2009). Functional characterization of TRPV4 as an osmotically sensitive ion channel in porcine articular chondrocytes. Arthritis Rheum.

[32] Goldsmith EJ, Rodan AR (2023). Intracellular Ion Control of WNK Signaling. Annu Rev Physiol.

[33] de Los Heros P, Alessi DR, Gourlay R (2014). The WNK-regulated SPAK/OSR1 kinases directly phosphorylate and inhibit the K+-Cl- co-transporters. Biochem J.

[34] Bertram KL, Banderali U, Tailor P (2016). Ion channel expression and function in normal and osteoarthritic human synovial fluid progenitor cells. Channels (Austin).

[35] Reed KSM, Ulici V, Kim C (2021). Transcriptional response of human articular chondrocytes treated with fibronectin fragments: an in vitro model of the osteoarthritis phenotype. Osteoarthritis Cartilage.

[36] Katsoula G, Lawrence JEG, Arruda AL (2024). Primary cartilage transcriptional signatures reflect cell-type-specific molecular pathways underpinning osteoarthritis. Am J Hum Genet.

[37] Gavile CM, Kazmers NH, Novak KA (2022). Familial Clustering and Genetic Analysis of Severe Thumb Carpometacarpal Joint Osteoarthritis in a Large Statewide Cohort. J Hand Surg Am.

[38] Jurynec MJ, Gavile CM, Honeggar M (2022). NOD/RIPK2 signalling pathway contributes to osteoarthritis susceptibility. Ann Rheum Dis.

[39] Jurynec MJ, Sawitzke AD, Beals TC (2018). A hyperactivating proinflammatory RIPK2 allele associated with early-onset osteoarthritis. Hum Mol Genet.

[40] Kazmers NH, Meeks HD, Novak KA (2021). Familial Clustering of Erosive Hand Osteoarthritis in a Large Statewide Cohort. *Arthritis Rheumatol*.

[41] Boyd-Shiwarski CR, Shiwarski DJ, Griffiths SE (2022). WNK kinases sense molecular crowding and rescue cell volume via phase separation. Cell.

[42] Yarikipati P, Jonusaite S, Pleinis JM (2023). Unanticipated domain requirements for Drosophila Wnk kinase in vivo. PLoS Genet.

[43] Goldring MB, Birkhead JR, Suen LF (1994). Interleukin-1 beta-modulated gene expression in immortalized human chondrocytes. J Clin Invest.

[44] Rinehart J, Vázquez N, Kahle KT (2011). WNK2 kinase is a novel regulator of essential neuronal cation-chloride cotransporters. J Biol Chem.

[45] Piala AT, Moon TM, Akella R (2014). Chloride sensing by WNK1 involves inhibition of autophosphorylation. Sci Signal.

[46] Yamada K, Park H-M, Rigel DF (2016). Small-molecule WNK inhibition regulates cardiovascular and renal function. Nat Chem Biol.

[47] Fisch KM, Gamini R, Alvarez-Garcia O (2018). Identification of transcription factors responsible for dysregulated networks in human osteoarthritis cartilage by global gene expression analysis. Osteoarthritis Cartilage.

[48] Ramos YFM, den Hollander W, Bovée JVMG (2014). Genes involved in the osteoarthritis process identified through genome wide expression analysis in articular cartilage; the RAAK study. PLoS One.

[49] Steinberg J, Ritchie GRS, Roumeliotis TI (2017). Integrative epigenomics, transcriptomics and proteomics of patient chondrocytes reveal genes and pathways involved in osteoarthritis. Sci Rep.

[50] Wang K, Esbensen QY, Karlsen TA (2021). Low-Input RNA-Sequencing in Patients with Cartilage Lesions, Osteoarthritis, and Healthy Cartilage. Cartilage.

[51] Adachi E, Katsumata O, Yamashina S (1999). Collagen II containing a Cys substitution for Arg-alpha1-519. Analysis by atomic force microscopy demonstrates that mutated monomers alter the topography of the surface of collagen II fibrils. Matrix Biol.

[52] Dudek M, Gossan N, Yang N (2016). The chondrocyte clock gene Bmal1 controls cartilage homeostasis and integrity. J Clin Invest.

[53] Snelling SJB, Forster A, Mukherjee S (2016). The chondrocyte-intrinsic circadian clock is disrupted in human osteoarthritis. Chronobiol Int.

[54] Yuan G, Xu L, Cai T (2019). Clock mutant promotes osteoarthritis by inhibiting the acetylation of NFκB. Osteoarthritis Cartilage.

[55] Cox KH, Takahashi JS (2019). Circadian clock genes and the transcriptional architecture of the clock mechanism. J Mol Endocrinol.

[56] Li X, Han Y, Li G (2023). Role of Wnt signaling pathway in joint development and cartilage degeneration. Front Cell Dev Biol.

[57] Sato A, Shimizu M, Goto T (2020). WNK regulates Wnt signalling and β-Catenin levels by interfering with the interaction between β-Catenin and GID. Commun Biol.

[58] Serysheva E, Berhane H, Grumolato L (2013). Wnk kinases are positive regulators of canonical Wnt/β-catenin signalling. EMBO Rep.

[59] Cao C, Shi Y, Zhang X (2022). Cholesterol-induced LRP3 downregulation promotes cartilage degeneration in osteoarthritis by targeting Syndecan-4. Nat Commun.

[60] Choi W-S, Lee G, Song W-H (2019). The CH25H-CYP7B1-RORα axis of cholesterol metabolism regulates osteoarthritis. Nature.

[61] Terkawi MA, Ebata T, Yokota S (2022). Low-Grade Inflammation in the Pathogenesis of Osteoarthritis: Cellular and Molecular Mechanisms and Strategies for Future Therapeutic Intervention. Biomedicines.

[62] Vincent TL (2020). Of mice and men: converging on a common molecular understanding of osteoarthritis. *Lancet Rheumatol*.

[63] Serysheva E, Mlodzik M, Jenny A (2014). WNKs in Wnt/β-catenin signaling. Cell Cycle.

[64] Yazici Y, McAlindon TE, Gibofsky A (2020). Lorecivivint, a Novel Intraarticular CDC-like Kinase 2 and Dual-Specificity Tyrosine Phosphorylation-Regulated Kinase 1A Inhibitor and Wnt Pathway Modulator for the Treatment of Knee Osteoarthritis: A Phase II Randomized Trial. *Arthritis Rheumatol*.

[65] Yazici Y, McAlindon TE, Gibofsky A (2021). A Phase 2b randomized trial of lorecivivint, a novel intra-articular CLK2/DYRK1A inhibitor and Wnt pathway modulator for knee osteoarthritis. Osteoarthritis Cartilage.

[66] Wang N, Lu Y, Rothrauff BB (2023). Mechanotransduction pathways in articular chondrocytes and the emerging role of estrogen receptor-α. Bone Res.

[67] Zhao Z, Li Y, Wang M (2020). Mechanotransduction pathways in the regulation of cartilage chondrocyte homoeostasis. J Cell Mol Med.

[68] Gossan N, Zeef L, Hensman J (2013). The circadian clock in murine chondrocytes regulates genes controlling key aspects of cartilage homeostasis. Arthritis Rheum.

[69] Hatzikotoulas K, Southam L, Stefansdottir L (2025). Translational genomics of osteoarthritis in 1,962,069 individuals. Nature.

