# Supplementary material for: WNK2 variants associated with familial osteoarthritis alter the chondrocyte response to hyperosmotic stress
Source: RMD Open. 2025 Jul 1;11(3):e005707. doi: 10.1136/rmdopen-2025-005707 (PMC12215121; doi:10.1136/rmdopen-2025-005707)
Supplement: online supplemental file 1 [file rmdopen-11-3-s001.pdf]

Supplementary information

METHODS

Study approval numbers

The study approval numbers are as follows: University of Utah (IRB # 79442), Intermountain Healthcare (IRB # 1050554), and University of Utah (IACUC protocol # 00001786).

Diagnostic and procedure codes used to identify individuals with osteoarthritis

Our coding strategy used to identify individuals with erosive hand, 1st MTP joint, distal and proximal interphalangeal joint, thumb joint, and shoulder osteoarthritis (OA) has been previously described.[1-3] Our study utilizes data drawn from the Utah Population Database (UPDB) (https://uofuhealth.utah.edu/huntsman/utah-population-database/). The UPDB provides person-based interlinked records documenting genealogy, medical records, and vital statistics for over 11 million individuals from the late 18th century to the present. We identify affected individuals and families from the UPDB using medical coding, which includes diagnostic and surgical procedure codes. To make sure we have a high degree of sensitivity and specificity, we initially identify affected individuals based on a specific form of OA (e.g., erosive hand OA, shoulder OA, interphalangeal joint OA, and 1st MTP joint OA) and then use statistical analyses to identify pedigrees that have a significant enrichment of that form of OA.[1-5]

The following diagnostic codes (ICD-9 and ICD-10) and procedure codes (CPT - Current Procedural Terminology) were used to identify affected individuals.

<u>1st MTP joint OA (synonymous with hallux rigidus):</u>

CPT: 28289 (hallux rigidus correction with cheilectomy, debridement and capsular release 1st metatarsophalangeal joint) and 28750 (arthrodesis, great toe).

ICD-9: 735.2 (hallux rigidus).

ICD-10: not used.

Erosive hand OA:

ICD-10 M15.4 (Erosive (osteo)arthritis).

Exclusion criteria:

Individuals diagnosed with any of the following codes were excluded:

ICD-9: 714.0 (rheumatoid arthritis), 714.2 and 714.3 (rheumatoid arthritis and other inflammatory polyarthropathies).

ICD-10: M05.xxx (rheumatoid polyneuropathy with rheumatoid arthritis), M06.xx (other rheumatoid arthritis), or M08.xxx (juvenile arthritis).

Individuals were asked if they were diagnosed with psoriatic arthritis, gout, or had a traumatic injury to the affected joint. If they answered yes, they were excluded from the study.

Sequence analysis of families with inherited OA

Families with high incidence of OA that appeared to segregate as a simple dominant Mendelian trait were identified.[1-3,6] Selected members were subjected to whole exome sequencing (WES) and candidate causative variants were identified.[2,6] Briefly, WES and analyses were performed using genomic DNA isolated from whole blood or saliva as previously described.[2,6] Libraries were prepared using the Agilent SureSelect XT Human All Exon + UTR (v8) kit followed by Illumina NovaSeq 6000 150 cycle paired end sequencing. We followed best practices established by the Broad Institute GATK for variant discovery (https://gatk.broadinstitute.org/hc/en-us).

Analysis of variants was performed with ANNOVAR[7] (http://annovar.openbioinformatics.org/en/latest/) and pVAAST[8] (http://www.hufflab.org/software/pvaast/) in concert with PHEVOR2[9] (http://weatherby.genetics.utah.edu/phevor2/index.html).

Statistical analysis

Statistical analyses were performed using GraphPad Prism 10 software (v10.3.1). All qPCR data was tested for normality using the Shapiro–Wilk test. Homogeneity of variances was tested using the Brown–Forsythe test between groups (ANOVA) and an F-test for paired samples (t-test). Any sample that failed the assumption of homogeneity of variance, was log transformed (Log_2) and retested. If the assumption of homogeneity of variance was not met after log transformation, a nonparametric Kruskal–Wallis test was used to test statistical significance.

qPCR analysis of *Wnk2* expression in the mouse knee joint: Statistically significant differences of $p \leq 0.05$ (*) were determined by one-way ANOVA with Tukey's multiple comparisons test, n=3 biological replicates.

qPCR analysis of OA-associated genes 5 days post-ACL rupture in the mouse knee joint: Statistically significant differences of $p \leq 0.05$ (*) were determined by a two-tailed unpaired t-test, n=4 biological replicates.

RNA-seq analysis: Differential gene expression was determined using median-ratio-normalization[10] with DESeq2 v1.30.0[11]. Fold-changes were calculated by comparing counts in all treatment groups normalized to the respective counterpart. DESeq2 utilizes Benjamini-Hochberg multiple testing correction within each comparison. Genes with an adjusted p value < 0.05 were considered differentially expressed, n=4 biological replicates.

qPCR analysis of gene expression in T/C-28a2 cells: Statistically significant differences of $p \leq 0.05$ (*) were determined by one-way ANOVA with Tukey's multiple comparisons test. n = 3-9 samples/condition. The *SLC12A2* (Supplementary Fig 8), *WNK2* (Supplementary Fig 6), and *ADAMTS-1* (Supplementary Fig 6) datasets were log transformed and statistically significant differences of $p \leq 0.05$ (*), $p \leq 0.01$ (**), and $p \leq 0.0001$ (****) were determined by a Kruskal–Wallis test.

qRT-PCR analysis of gene expression in primary human chondrocytes: Statistically significant differences of $p \leq 0.05$ (*) were determined by a two-tailed unpaired t-test, n=4 biological replicates. *ADAMTS1* (Supplementary Fig 9)and *PER1* (Supplementary Fig 10) datasets were log transformed and statistically significant differences of $p \leq 0.05$ (*) were determined by one-way ANOVA with Tukey's multiple comparisons test.

Isolation of RNA from mouse knee joints

Mice were euthanized, the skin was removed from the hindlimb, and the entire limb was placed in RNAlater solution (ThermoFisher Scientific). The knee joint was exposed by removing the majority of muscle surrounding the joint with minimal disruption of the ligaments, tendons, synovium, and infrapatellar fat pad. The femur and tibia were cut approximately 0.5 - 1 cm distal from the articular surface. The joint was then transferred to new RNAlater solution, rinsed, cut into small fragments, transferred to a tube containing Trizol and 2.8mm stainless steel beads, and homogenized using the BeadBug microtube homogenizer. Total RNA was isolated using Direct-zol RNA Miniprep Kit (Zymo Research) and quantified using The Agilent TapeStation.

Generation of *WNK2* null T/C-28a2 chondrocytes using CRISPR/Cas9 genome editing

WNK2 null (*WNK2*$^{-}$) T/C-28a2 chondrocytes (MilliporeSigma, SCC042) were generated by the Genomics Core Facility at the University of Utah. Briefly, electroporation of CRISPR/Cas9 ribonucleoproteins targeting the introns surrounding exon 2 of *WNK2* were used to generate a deletion of exon 2 (Fig. 2A and Supplementary Fig. 3). Following electroporation T/C-28a2 chondrocytes were grown for three passages, single clones were isolated by flow cytometry, and clonal lines were established. Deletion of exon 2 was verified by PCR and Sanger sequencing (Supplementary Table 5 and Supplementary Fig. 3).

Preparation of cell lysates and immunoblot analysis

To test if *WNK2*$^{-}$ chondrocytes lack WNK2 function, WT and *WNK2*$^{-}$ chondrocytes were cultured as described above and the cells were subjected to osmotic stress as previously described.[12] Briefly, once chondrocytes reached 70% confluence, they were replaced with fresh complete medium (isotonic/300mOsm) or with sorbitol (50mM/350mOsm, 200mM/500mOsm, and 500mM/800mOsm). For WNK inhibitors experiments, WT chondrocytes were preincubated for 15 minutes with 100µM WNK463 (MedChemExpress, HY-100626) and treated with sorbitol as described below. WNK46 is a pan-WNK (WNK1-4) inhibitor with IC_{50s} of 5nM, 1nM, 6nM, and 9nM for WNK1, WNK2, WNK3, and WNK4, respectively.[13] Chondrocytes were incubated for five minutes in an isotonic or stress-inducing medium and harvested and pelleted at 4°C. The total exposure of osmotic stress was 15 minutes. Proteins were isolated using RIPA buffer supplemented with protease and phosphatase inhibitors. The isolated proteins were separated by sodium dodecyl sulfate-polyacrylamide gel electrophoresis (SDS-PAGE) using a 10% acrylamide gel and transferred to a PVDF (0.45um) membrane. The membrane was blocked with 3% BSA in Tris-buffered saline with 0.1% Tween-20 (TBST) overnight at 4°C. The membranes

were incubated overnight at 4°C with primary antibodies against SPAK (Abcam ab192798) or pSPAK antibodies (EMD Millipore, 07-2273). Blots were washed 3X with TBST and then incubated with secondary antibodies conjugated with horseradish peroxidase (anti-mouse/rabbit IgG HRP, ThermoFisher Scientific). Signal detection was achieved through enhanced chemiluminescence (SuperSignal West Pico Chemiluminescent Substrate 34080, ThermoFisher Scientific). GAPDH (Santa Cruz Biotechnology, 365062) served as the loading control. The bands were quantified using ImageJ by measuring the Integrated Density (Area × Mean Gray Value) of each band. The values were normalized to the Iso treatment within their respective groups for relative quantification.

Immunohistochemistry and Safranin O and Fast Green staining

Human tissue - Human humeral heads were collected during total shoulder arthroplasties at the University of Utah Orthopaedic Center. Tissue was collected directly into 10% neutral buffered formalin and incubated at 4ºC for 10 days, processed through an ethanol series, demineralized using FormiCal, equilibrated in xylenes, and embedded in paraffin. 5-6 µm thick tissue sections were cut and mounted onto slides. For histological analysis, slides were stained with Fast Green for 2 minutes and rinsed with acetic acid. Slides were then stained with Safranin-O for 8 minutes and dehydrated with sequential washings of 95% ethanol (EtOH) and 100% EtOH. Slides were fixed with Histochoice for 4 minutes (2X) and then mounted. Mouse tissue - Animals were euthanized and knees were dissected and fixed in 10% neutral buffered formalin at 4ºC for 48 hours, processed through an ethanol series, demineralized using FormiCal, equilibrated in xylenes, and embedded in paraffin. 5-6 µm thick tissue sections were cut and mounted onto slides. All tissue sections were deparaffinized and rehydrated as follows: Xylene 3X 5 minutes,

95% EtOH 2X 3 minutes, 70% EtOH 2X 3 minutes, ddH_2O 1X 2 minutes, and 1X PBS for 5 minutes. For immunohistochemistry, antigen retrieval was according to the antibody manufacturer's protocol. We utilize the following primary antibodies: WNK2 (Abcam, ab239037), SPAK (Abcam, ab192798), and pSPAK (EMD Millipore, 07-2273). Tissue sections were incubated with primary antibody at 4ºC, for 18-24 hours in a humidified chamber, rinsed 3X with PBS, and then blocked with 2.5% horse serum (Vector Laboratories, MP-7401) for 20 minutes at 25ºC. Control tissues sections (mouse and human) were not incubated with primary antibody and processed with the appropriate secondary antibody. Block solution was replaced with ImmPRESS-HRP Horse anti-rabbit/mouse secondary antibody (Vector Laboratories, MP-7401/ MP-7402-15) and incubated for 30 minutes at room temperature followed by 2X 5 minutes rinses with PBS. Slides were stained with DAB (Vector Laboratories, SK-4100) for 10-20 minutes, rinsed for 5 minutes with PBS, dried, and mounted.

Anterior cruciate ligament rupture

Non-invasive ACL rupture was induced by a single overload cycle of tibial compression as previously described.[2,14] Sixteen-week old male mice were used in all ACL rupture experiments.

T/C-28a2 chondrocyte cell culture

The human chondrocyte cell line, T/C-28a2 (MilliporeSigma, SCC042), was cultured in Dulbecco's modified Eagle's medium (DMEM) (Corning Life Sciences) supplemented with 10% fetal bovine serum (FBS; Avantor), 4.5 g/L glucose, 1mM sodium pyruvate, and 100 U/mL penicillin/streptomycin in a 5% CO_2 humidified atmosphere incubator at 37°C. Cell passage was

performed on 70% confluent cells by trypsinization with 0.25% trypsin-EDTA (ThermoFisher Scientific).

Transfection and treatment of T/C-28a2 chondrocytes

Twelve hours after seeding 200 × 10^3 WT or WNK2-deficient T/C-28a2 chondrocytes in T-25 tissue culture plates, the cells were transfected with 2µg of WNK2 expression plasmids using Lipofectamine 2000 (Thermo Fisher Scientific). After 48 hours, transfection media was replaced with fresh DMEM media supplemented with 2% fetal bovine serum, 4.5 g/L glucose, 1mM sodium pyruvate, and 100 U/mL penicillin/streptomycin containing varying amounts of sorbitol for an additional 7 days. Sorbitol was added to the medium to alter osmolarity [12] as WNKs are inhibited by Cl^- ions.[15] After incubation cells were collected by trypsinization, snap frozen, and RNA expression was analyzed (described below). To ensure equal transfection between replicates and experiments, we determined the normalized read counts for *WNK2*.

Quantitative PCR (qPCR)

To optimize the sorbitol dose**,** $5x10^3$ T/C-28a2 chondrocytes (MilliporeSigma, SCC042),were plated in T-25 plates, after 12 hours (settling time), cells were treated with 25, 50, 100, and 150mM sorbitol, no treatment was considered as isotonic, in DMEM + 2% FBS with antibiotics and incubated for 7 days at 37°C. After incubation, cells were lysed using Trizol (ThermoFisher Scientific), and RNA was collected using Direct-zol RNA Miniprep Kits (Zymo Research), and RNA quantity and purity was assessed using the Nanodrop 2000 (ThermoFisher Scientific) and The Agilent TapeStation. 500µg of total RNA was reverse transcribed using the Maxima First Strand cDNA Synthesis Kit (ThermoFisher Scientific). Gene-specific primers (Supplementary

Table 5) were used to amplify cDNA using Luna Universal qPCR Master mix (New England BioLabs). 3-6 biological replicates were used in each treatment group. Gene expression was normalized to *β-ACTIN*, and the relative expression was estimated using the ΔΔCq method.[16] Fold-change values were analyzed without log transformation. The mNA levels are expressed as fold-change mean with 95% confidence interval relative to isotonic conditions.

Generation of *WNK2* mutant constructs

WT and mutant *WNK2* constructs were synthesized using GenScript's CloneEZ service. The WT *WNK2* construct was derived from a plasmid sequence deposited in Addgene (Catalog #24569) and subsequently modified by GenScript to generate the OA-associated variants, $WNK2^{H758N}$, $WNK2^{L1005F}$, and $WNK2^{R2054Q}$. All clones were verified by Sanger sequencing.

Primary human chondrocyte cell culture

Primary human chondrocytes (CELLvo™ HC-a (P1)) were obtained from StemBioSys, Inc. CELLvo™ Chondrocytes were isolated from cadaveric articular cartilage of healthy donors (<40 years old). CELLvo™ HC-a chondrocytes were cultured on CELLvo™ ChondroMatrix coated plasticware (StemBioSys, Inc.) in low glucose Dulbecco's Modification of Eagle's Medium (DMEM) (Corning) supplemented with 1 g/L glucose, 1mM sodium pyruvate, 4 mM L-glutamine, 15% fetal bovine serum and 100 U/mL penicillin/streptomycin in a 5% CO_2 humidified atmosphere incubator at 37°C. Cell passage was performed on 70% confluent cells by trypsinization with 0.05% trypsin-EDTA (ThermoFisher Scientific).

Electroporation of primary human chondrocytes

Electroporation of CELLvo™ HC-a chondrocytes was carried out with the Neon™ Transfection System using the 10µL Kit (ThermoFisher Scientific) following the manufacturer's protocol. Briefly, 200 x 10^3 CELLvo™ HC-a chondrocytes and 2ug of *WNK2* or *WNK2*^{R2054Q} plasmids suspended in buffer R were electroporated using parameters the following parameters: 1600 V, 10 msec, 5 pulse. These conditions resulted in >95% electroporation efficiency as assayed by the total number of GFP^+ cells (data not shown). Cells were then transferred into a 6-well flask with 5 ml of pre-warmed media. After 48 hours, transfection media was replaced with fresh low glucose DMEM media supplemented with 2% fetal bovine serum, 4.5 g/L glucose, 1mM sodium pyruvate, 4 mM L-glutamine, and 100 U/mL penicillin/streptomycin containing varying amounts of sorbitol for an additional 7 days. After incubation cells were collected by trypsinization, RNA was isolated and analyzed by qRT-PCR as described below.

Quantitative Reverse Transcription PCR (qRT-PCR)

Electroporated primary CELLvo™ HC-a chondrocytes were lysed using Trizol (ThermoFisher Scientific), and total RNA was collected using the Direct-zol RNA Miniprep Kit (Zymo Research). RNA quantity and purity was assessed using the Nanodrop 2000 (ThermoFisher). A total of 50ng of RNA was combined with PrimeTime Master Mix (IDT) and gene-specific primers (IDT; see below) and qRT-PCR was performed according to the manufacture's protocol. Gene expression was normalized to *ACTB*, and relative expression was calculated using the ΔΔCq method. mRNA levels are reported as Fold-Change (mean with 95% confidence interval) relative to each control condition. Statistically significant differences of $p \leq 0.05$ (*) and $p \leq 0.01$ (**) were determined by a two-tailed unpaired t-test, n=4 biological replicates.

	Gene Name	Assay ID
1	*ACTB*	Hs.PT.39a.22214847
2	*ACAN*	Hs.PT.56a.742783
3	*SOX9*	Hs.PT.58.38984663
4	*CCL2*	Hs.PT.58.45467977
5	*POSTN*	Hs.PT.58.4452022
6	*ADAMTS1*	Hs.PT.58.1452444
7	*ADAMTS3*	Hs.PT.58.4659383
8	*PER1*	Hs.PT.58.3826844
9	*CRY1*	Hs.PT.58.45565891
10	*NFIL3*	Hs.PT.58.4948721
11	*NPAS2*	Hs.PT.58.1532958

RNA-sequencing and data analysis

WT, *WNK2*$^{-}$, or transfected T/C-28a2 chondrocytes (5x10^3 cells) were plated in T-25 tissue culture plates and treated as described above. All RNA-seq experiments were performed in quadruplicate (See PCA plot Supplementary Figure 7). RNA extraction, quality control, and RNA-sequencing (RNA-seq) was performed by Novogene. Data analysis was performed in house. Aligned reads were counted using HTSeq v0.11.3[17]. We performed several different analyses in DESeq2 v1.30.0[11]. First, we combined all the factors (samples, treatment, and

genotype) and ran DESeq2 to obtain the regularized logs (rlog) for sample visualizations. The rlog values are similar to log2 normalized counts except the variance in low count genes is reduced. We then plotted the first two principal components using the rlog values from the top 500 variable genes (Supplementary Figure 7). The PCA plot indicates that there is minimal variation between samples and therefore dispersion is not inflated. We then combined all 48 samples and then ran pairwise comparisons. We then performed 2 interaction models. One with genotype vs treatment (model 1) and the other with transfection vs treatment (model 2). We then created interaction plots with the top 25 genes (Supplementary Figure 7). An interaction model will test if the slopes of these lines (log2 fold changes) are different.

The raw RNA-seq counts were then analyzed for differential gene expression using median-ratio-normalization[10] with DESeq2 v1.30.0[11]. Fold-changes were calculated by comparing counts in all treatment groups normalized to the respective counterpart. DESeq2 utilizes Benjamini-Hochberg multiple testing correction within each comparison. Genes with an adjusted p value < 0.05 were considered differentially expressed. RNA-seq datasets are deposited on the Gene Expression Omnibus (GSE244584). *WNK2* expression levels were comparable between transfected cells as analyzed by comparison of normalized read counts from RNA-seq data (Supplementary Table 4).

Identification of differentially expressed genes in previously published osteoarthritis RNA-seq datasets

We identified 80 up- and 52 down-regulated DEGs from five independent previously published five independent studies using RNA-seq and microarray technologies, focusing on knee and hip

cartilage from human osteoarthritis tissues (Supplementary Table 6).[18-22] We included genes if they had a p value less than 0.05 and were present in at least three studies. These included DEGs in primary chondrocytes from normal human femoral cartilage treated with fibronectin (FN-f)[20], preserved cartilage from OA-affected hip and knee joints versus healthy cartilage[19], paired intact and degraded articular cartilage from 38 patients undergoing joint replacement surgery (12 knee OA, 17 knee OA, 9 hip OA)[21], cartilage biopsies from OA patients undergoing knee replacement compared to healthy cartilage from ACL-reconstruction patients[22], and 18 normal versus 20 OA human knee cartilage tissues.[18]

Family	OA Phenotypes and Individuals Analyzed
**MTP24**	1st MTP Joint OA - 2 affected, 1 unaffected. Proband (F), sister (affected), and brother (unaffected). Affected individuals were diagnosed with bilateral 1st MTP joint OA. Proband has 1st MTP joint bone erosions. Sister also diagnosed with 2nd and 3rd MTP joint OA.
**ERO32**	Erosive hand OA - 4 affected (F) and 4 unaffected (F). Proband, 2 sisters, and one daughter affected. 3 daughters and niece unaffected. Affected individuals were diagnosed with bilateral erosive hand OA. Proband was also diagnosed with spine, bilateral knee, and shoulder (right) OA. One sister was diagnosed with bilateral knee OA. One sister was diagnosed with hip, bilateral knee and spine OA. The daughter was diagnosed with bilateral wrist, elbow, hip, knee, and foot (left) OA.
**ERO549024**	Erosive hand OA - 3 affected (1F and 2M), 1 unaffected (M). Proband, son, and distant cousin affected. One son unaffected. All affected individuals were diagnosed with bilateral erosive hand OA. Son was also diagnosed with wrist OA (left).
**ERO20**	Erosive hand OA - 3 affected (F) and 1 unaffected (M). Proband, sister, and daughter affected. Son unaffected. Affected individuals were diagnosed with bilateral erosive hand OA. Proband was also diagnosed with bilateral thumb and left wrist OA. The daughter was diagnosed with neck, shoulder, elbow, and knee OA. Sister diagnosed with neck, knee, and elbow OA.

Supplementary Table 1. OA Families and Phenotype Details

Log2 Fold Change (adj. P. value)	Accession Number	Species	Tissue	Type	Description and Comparison	Reference
1.19	GSE121033	Mus musculus	Cartilage	RNA seq	Polycomb protein 2 overexpression in ACLT induced osteoarthritis; shams vs ACLT induced osteoarthritis	Not yet published
0.301 (0.079)	GSE51588	Homo sapiens	Bone	Microarray	Subchondral Bone in Osteoarthritis; Subchondral bone from medial tibial vs Normal-Subchondral bone from medial tibial	[23]
0.204 (0.096)	GSE13837	Homo sapiens	Synovium	Microarray	TNFa treated synovium from rheumatoid arthritis or osteoarthritis; TNF stimulated synovial fibroblasts (4h).rheumatoid arthritis vs TNF stimulated synovial fibroblasts (4h).osteoarthritis	[24]
-0.348	GSE60162	Homo sapiens	Synovium	Microarray	Osteoarthritis synovial fibroblasts transfected with TBX5; TBX5 overexpression vs vector control	[25]
-0.458 (0.979)	Cytokine Explant	Homo sapiens	Cartilage	RNA seq	IL1B or TNF induced expression in osteoarthritis cartilage explants; IL1 vs TNF	Not yet published
-0.846 (0.188)	Cytokine Explant	Homo sapiens	Cartilage	RNA seq	IL1B or TNF induced expression in osteoarthritis cartilage explants; TNF vs Control	Not yet published
-1.18 (0.112)	Cytokine Explant	Homo sapiens	Cartilage	RNA seq	IL1B or TNF induced expression in osteoarthritis cartilage explants; DMC vs DLC	Not yet published
-1.3 (0.012)	Cytokine Explant	Homo sapiens	Cartilage	RNA seq	IL1B or TNF induced expression in osteoarthritis cartilage explants; IL1 vs Control	Not yet published
-1.32 (0.013)	Cytokine Explant	Homo sapiens	Cartilage	RNA seq	IL1B or TNF induced expression in osteoarthritis cartilage explants; Control vs DLC	Not yet published

Supplementary Table 2. *WNK2* expression in SkeletalVis datasets.[26]

Ensembl ID	Gene	Normalized counts from RNA-seq analysis<br>WT T/C-28a2 cells	 *WNK2*$^-$ T/C-28a2 cells
ENSG00000060237	WNK1	6293.41	7832.92
ENSG00000165238	WNK2	2443.34	398.62
ENSG00000196632	WNK3	250.85	210.42
ENSG00000126562	WNK4	406.75	575.54

Supplementary Table 3. *WNK1-4* mRNA expression in WT and *WNK2*$^-$ T/C-29a2 chondrocytes. There is no statistically significant upregulation of *WNK1*, *WNK3*, or *WNK4* in *WNK2*$^-$ cells.

Ensembl ID	Gene	Normalized read counts from RNA-seq analysis					
		WT T/C-28a2 cells				*WNK2*⁻ T/C-28a2 cells	
		Ctr	Sor	*WNK2* trans	*WNK2* trans +Sor	Ctr	Sor
**ENSG00000165238**	WNK2	2443.34	2936.97	2444.01	2972.89	398.62	354.31

Supplementary Table 4. *WNK2* mRNA expression levels in WT and *WNK2*$^-$ T/C-29a2 chondrocytes after various treatments. There is no statistically significant change in *WNK2* expression after sorbitol (Sor) treatment or *WNK2* transfection (*WNK2* trans). However, there is significant decrease in *WNK2* expression in *WNK2*$^-$ cells compared with WT.

Gene	Species	Primer Sequence (5'-3')
GAPDH	*Homo sapiens*	FWD:TCG GAG TCA ACG GAT TTG GT<br>RVS:TTC CCG TTC TCA GCC TTG AC
ACTB	*Homo sapiens*	FWD:GCC GCC AGC TCA CCA T<br>RVS:TCG TCG CCC ACA TAG GAA TC
COL2A1	*Homo sapiens*	FWD:ATG AGG GCG CGG TAG AGA<br>RVS:GCC AGC CTC CTG GAC ATC
MMP13	*Homo sapiens*	FWD:AGC CTT CAA AGT TTG GTC CGA<br>RVS:AGA AGT CGC CAT GCT CCT TA
ADAMTS1	*Homo sapiens*	FWD:GCG TCA ATG CTT TCC AAC CTG G<br>RVS:GGG ATT CTG AGG CTT GTC CAT C
ADAMTS5	*Homo sapiens*	FWD:TGG CTC ACG AAA TCG GAC ATT<br>RVS:TGC ATT TGG ACC AGG GCT TA
SOX9	*Homo sapiens*	FWD:AGG AAG CTC GCG GAC CAG TAC<br>RVS:GGT GGT CCT TCT TGT GCT GCA C
WNK2	*Homo sapiens*	FWD:ACG CAC CCG ATG AAA TTG CCA C<br>RVS:TCT GTG TCC TCG CTG AGC ATG T
SLC12A2	*Homo sapiens*	FWD:CCT CTA CAC AAG CCC TGA CTT A C<br>RVS:CGT GAG TTT GGA GCA CCT GTC A
WNK2-exon2	*Homo sapiens*	FWD:GAC CTC GCC CGG AAC TC<br>RVS:CAC TTT TGG CAA ATG ACG CTC T
Wnk2	*Mus musculus*	FWD:CTG ATG GTG GAA GTG GCT CAA G<br>RVS:CTC CAA AGG TGC CTT CAC AGC T
Mmp3	*Mus musculus*	FWD:TTG TGT GCT CAT CCT ACC CAT T G<br>RVS:TTC CTC CAT TTT GGC GAA CC
Tnfα	*Mus musculus*	FWD:ATG AGC ACA GAA AGC ATG ATC<br>RVS:TAC AGG CTT GTC ACT CGA ATT
Il1β	*Mus musculus*	FWD:TCC CAA GCA ATA CCC AAA GAG A A<br>RVS:TGG GGA AGG CAT TAG AAA CAG T C
iNos	*Mus musculus*	FWD:GCA TGG ACC AGT ATA AGG CAA G CA<br>RVS:GCT TCT GGT CGA TGT CAT GAG C AA

Supplementary Table 5. qPCR and PCR primer sequences used in this study.

Up-regulated DEGs		Down-regulated DEGs	
SLC7A2	*ATP1B1*	*NR1H3*	*KBTBD11*
BAIAP2L1	*TIAM2*	*SYNE2*	*SHMT1*
CD44	*IGFBP3*	*ATP9A*	*C11orf71*
BMAL2	*LRP12*	*GSTO2*	*IRS2*
RAI14	*PTGES*	*TLE2*	*RXRA*
WWC3	*ADD3*	*ACACB*	*ZNF33B*
PFKP	*MFSD6*	*ZNF446*	*UAP1L1*
FSTL3	*PLOD2*	*SLC7A8*	*WWP2*
WDR1	*PID1*	*CCNB1IP1*	*NHSL2*
CA12	*ABI3BP*	*MFAP2*	*VIT*
SEMA3C	*TAGLN2*	*RCOR3*	*SCARF2*
PLD1	*ABR*	*EEPD1*	*CLDN23*
AKR1B1	*TGFA*	*SH3BP4*	
MID1	*FSTL1*	*PNPLA7*	
PGK1	*IGFBP7*	*GSE1*	
SLC7A5	*TNFRSF11B*	*PRKAB2*	
SLC39A14	*ANGPTL4*	*ITGB4*	
KCNN4	*COL3A1*	*BEX2*	
CDK6	*MT1E*	*PSRC1*	
NPTX2	*TM4SF1*	*MNS1*	
HSPA8	*CDH2*	*TTYH2*	
CCND1	*PGAM1*	*ALDH1L1*	
TPI1	*P2RY6*	*PIEZO2*	
SLC16A10	*BPGM*	*COLEC12*	
WNT5A	*BASP1*	*DHRS3*	
COL7A1	*ARSJ*	*CDC42EP3*	
SLC2A1	*GINS3*	*FZD5*	
PRDX1	*PAPPA*	*PTPN13*	
RARRES1	*AMTN*	*ARHGEF3*	
HSPH1	*DNER*	*CRYL1*	
SRGN	*CD55*	*NDRG2*	
TNFAIP6	*LAMB3*	*GPT2*	
MT2A	*PDLIM7*	*PLEKHF1*	
TTC9	*HNRNPAB*	*RCOR2*	
DYSF	*COL13A1*	*RAB3IL1*	
KCNK1	*MT1F*	*PLEKHA2*	
SERPINE2	*PAPSS2*	*DCXR*	
LRRC1	*PPP1R14C*	*TLN2*	
SLC39A8	*TGM2*	*FZD4*	
SLC16A3	*TENM3*	*SYNE3*	

Supplementary Table 6. 80 up-regulated and 52 down-regulated differentially expressed genes (DEGs) derived from five independent, previously published RNA-seq datasets of human osteoarthritis tissues.

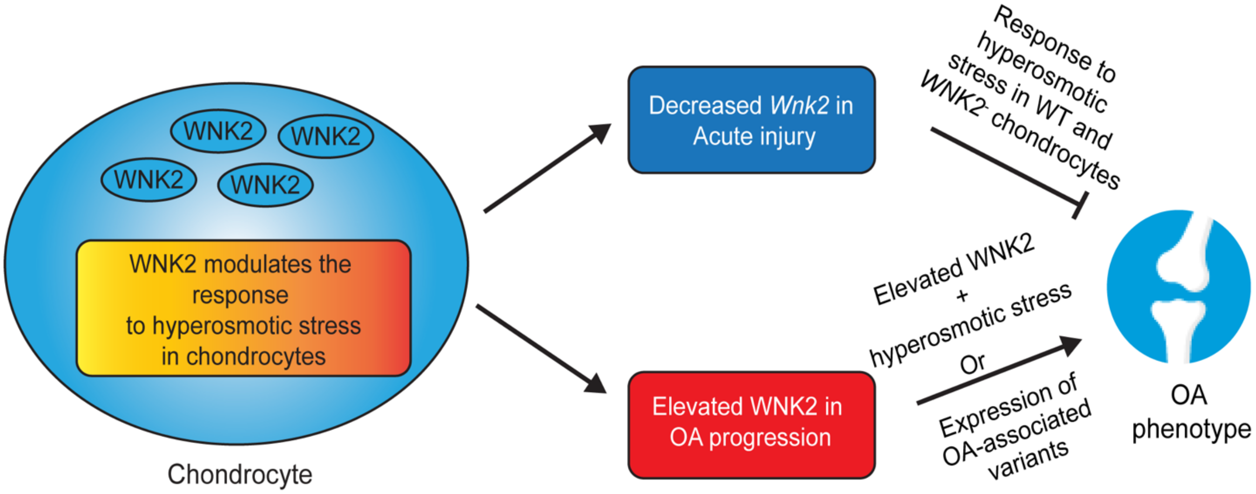

Supplementary Fig. 1 Graphical abstract illustrating the proposed role of WNK2 activity in chondrocytes and its contribution to osteoarthritis (OA) pathogenesis. The diagram illustrates the dynamic regulation of WNK2 and how alterations in its expression, combined with hyperosmotic stress, contribute to osteoarthritis (OA) progression.

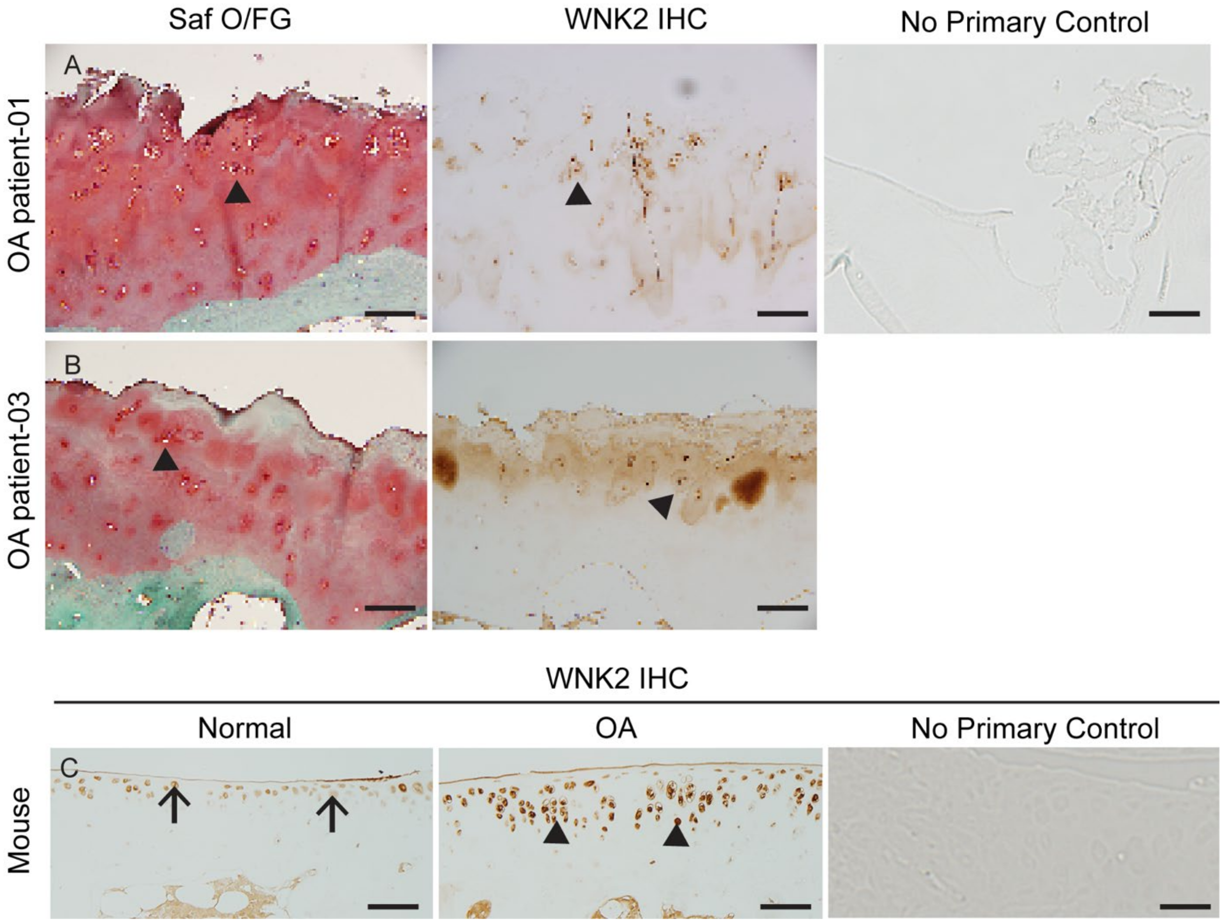

Supplementary Fig. 2 WNK2 expression is elevated in chondrocytes present in injured human osteoarthritic tissue and mouse joints. (A-B) Representative images of humeral head cartilage isolated from OA patients stained with Safranin O and Fast Green (Saf O/FG) and immunohistochemical staining for WNK2 expression. Arrowheads mark regions with severely damaged cartilage and overexpressed WNK2 in hypertrophic chondrocytes. (C) Immunohistochemical staining demonstrating WNK2 expression in uninjured (Normal) and in hypertrophic chondrocytes of the mouse tibia 8 weeks post injury (OA). No primary antibody controls have no positive signal in human or mouse tissue. Scale bar = 100µm.

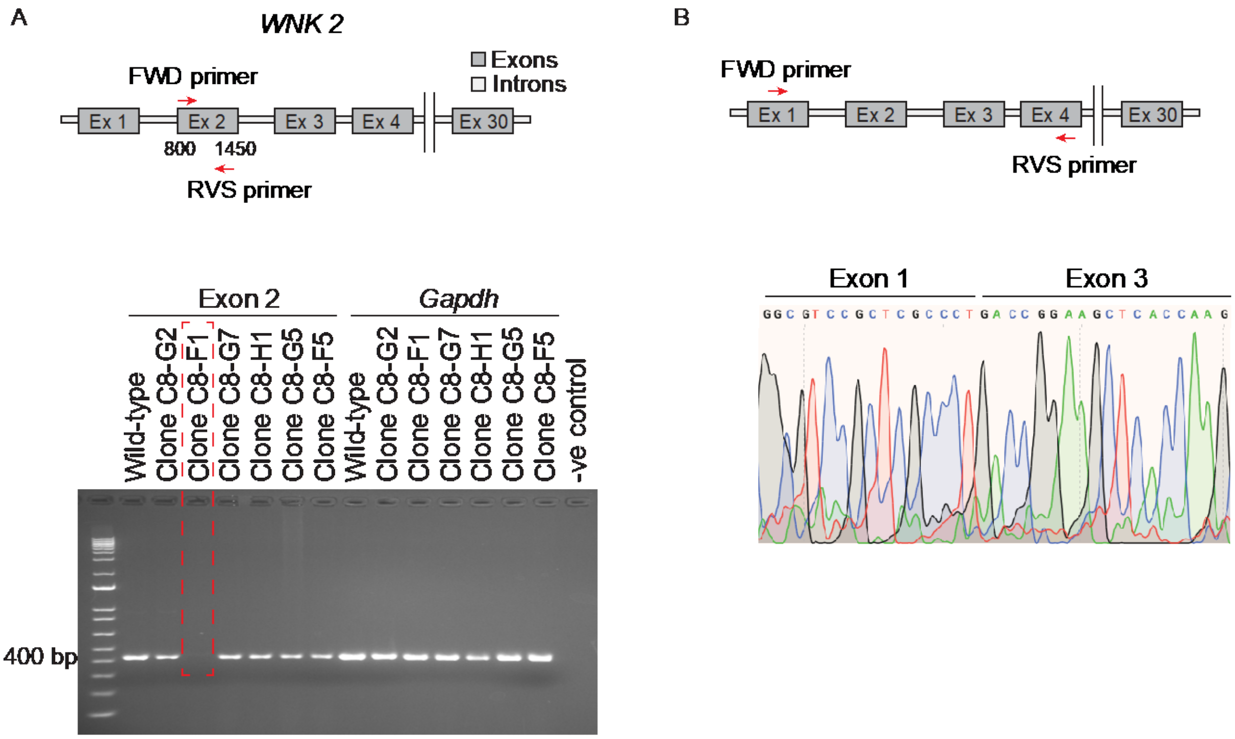

Supplementary Fig. 3 Generation and validation of *WNK2*$^{-}$ T/C-28a2 chondrocytes. (A) Schematic illustration of the human *WNK2* locus. Primers used to amplify exon 2 are indicated as red arrows. Agarose gel image depicting PCR amplification using DNA isolated from putative mutant clones. Wild-type indicates DNA isolated from unmodified control chondrocytes. *GAPDH* PCR amplification is used as a control. (B) Schematic illustration of the human *WNK2* locus indicating primers (red arrows) used to amplify *WNK2* from control and clone C8-F1 cDNA. This amplicon was subjected to Sanger sequencing and the chromatogram indicates deletion of exon 2 from clone C8-F1 cDNA, which removes the nascent start codon.

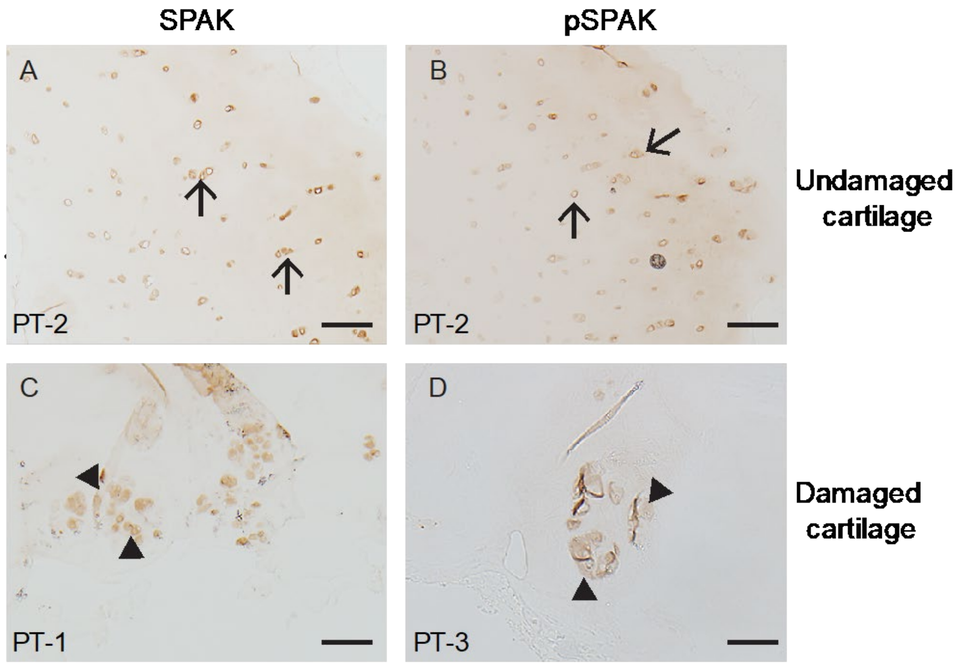

Supplementary Fig. 4 SPAK and pSPAK are expressed in undamaged and damaged human osteoarthritic tissue. (A-C) Representative images of humeral head cartilage isolated from OA patients immunostained for SAPK (A and C) and pSPAK (B and D). Arrows indicate SPAK and pSPAK expression in chondrocytes in undamaged cartilage. Arrowheads mark SPAK and pSPAK expression in severely damaged cartilage. Scale bar = 100µm.

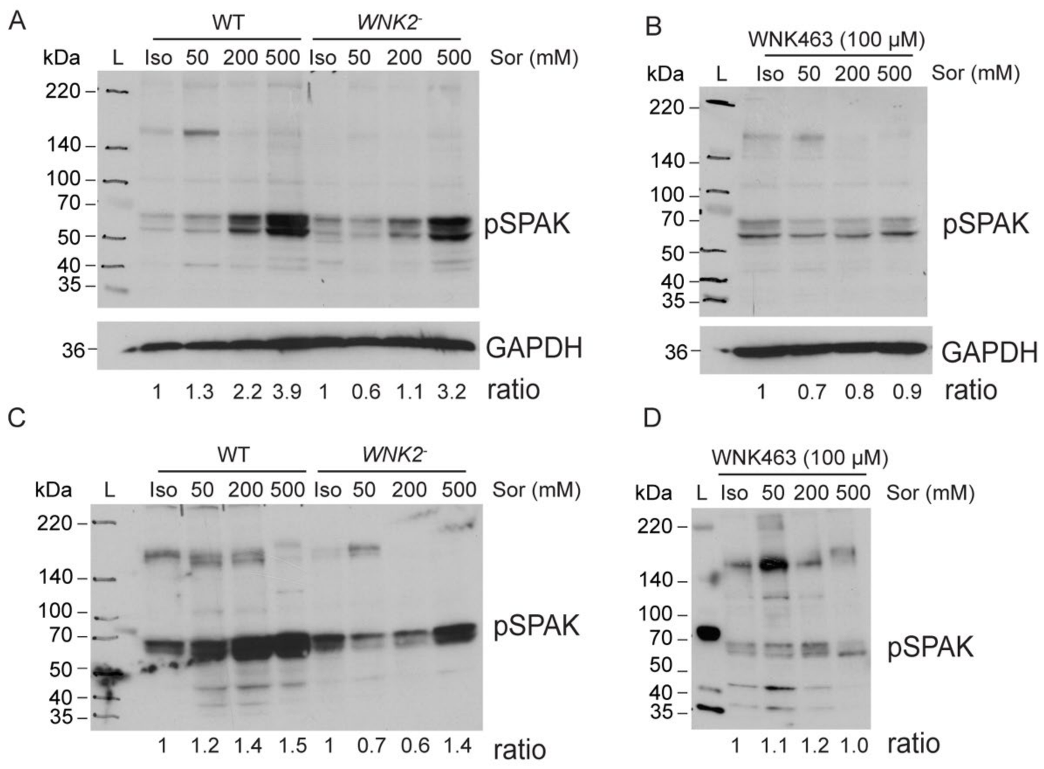

Supplementary Fig. 5 WNK2 mediates the acute response to hyperosmotic stress in chondrocytes. (A) pSPAK levels in WT and *WNK2*- chondrocytes exposed to increasing levels of hyperosmotic stress. (B) pSPAK levels in WT chondrocytes exposed to hyperosmotic stress in the presence of a WNK1-4 inhibitor (WNK463, 100µM). Both blots are unmodified/uncropped. (C and D) Unmodified/uncropped immunoblots used to generate Fig. 2B and C. Ratio indicates the levels of pSPAK in each treatment relative to isotonic conditions. GAPDH is used as a loading control.

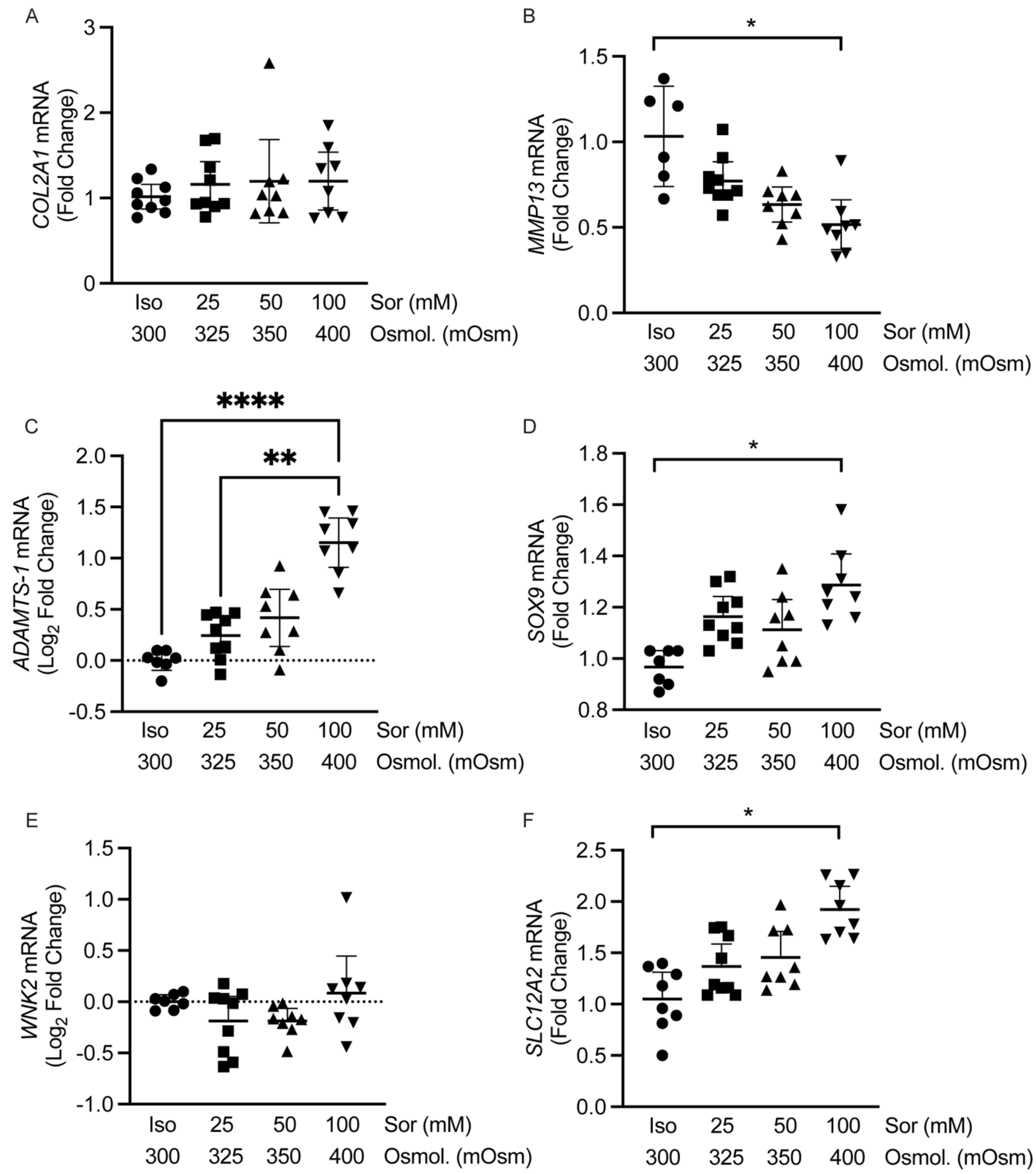

Supplementary Fig. 6 Dose response of hyperosmotic stress in T/C-28a2 human chondrocytes. T/C-28a2 human chondrocytes were treated with 25, 50, and 100 mM sorbitol (Sor). No treatment was considered isotonic (Iso). (A-D) qPCR analysis of the OA-associated genes, *COL2A1, MMP13, ADAMTS-1* and *SOX9*; qPCR analysis of *WNK2* (E) and *SLC12A2* (F). Data are expressed as Fold Change (mean with 95% confidence interval) relative to Iso treated T/C-28a2 human chondrocytes. n = 6-9 samples/condition. Error bars represent 95% confidence

interval and a statistically significant difference of $p \leq 0.05$ (*) was determined by one-way ANOVA with Tukey's multiple comparisons test, except for *WNK2* and *ADAMTS-1*, which statistically significant differences of $p \leq 0.05$ (*), $p \leq 0.01$ (**), and $p \leq 0.0001$ (****) were determined by a Kruskal–Wallis test.

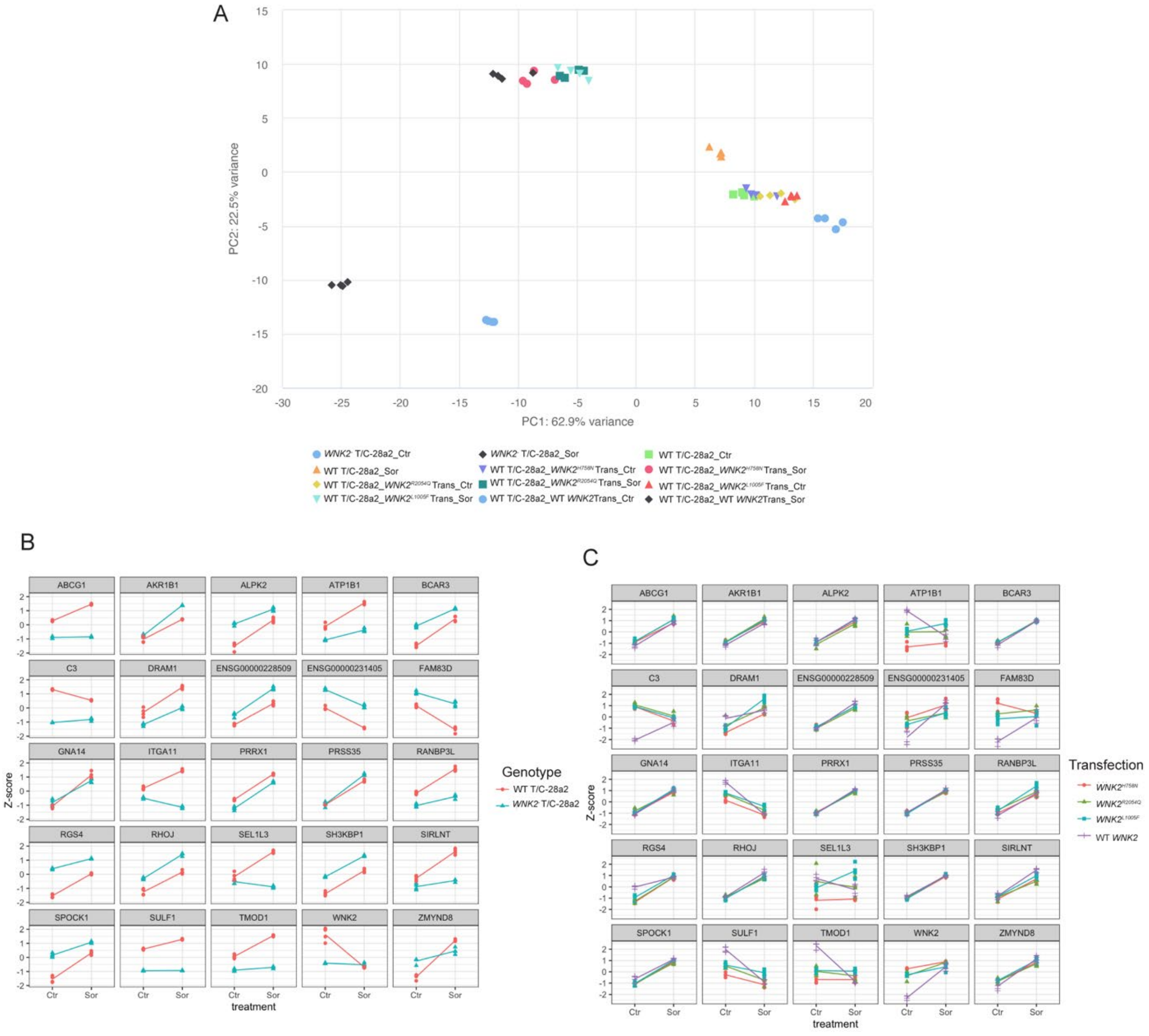

Supplementary Fig. 7 PCA plot and interaction analysis of gene expression across treatments. Comparative analysis of RNA-seq performed on chondrocytes transfected with WT *WNK2* or *WNK2* OA-associated variant plasmids (48-hour transfection) and treated with 100mM sorbitol (Sor) for 7 days. (A) PCA plot of 48 samples across 12 treatments, using the first two principal components derived from the rlog-transformed values of the top 500 most variable genes. (B) Interaction model 1: The top 25 interaction terms between "treatment" (with and without sorbitol) and "genotype" (WT and *WNK2*$^{-}$ chondrocytes), with Z-scores representing the standardized effect size of these interactions. (C) Interaction model 2: The 25 interaction terms between "transfection" (WT-*WNK2*, *WNK2*^{H758N}, *WNK2*^{R2054Q}, or *WNK2*^{L1005F}) and "treatment", where Z-scores indicate the direction and magnitude of combined effects on gene expression.

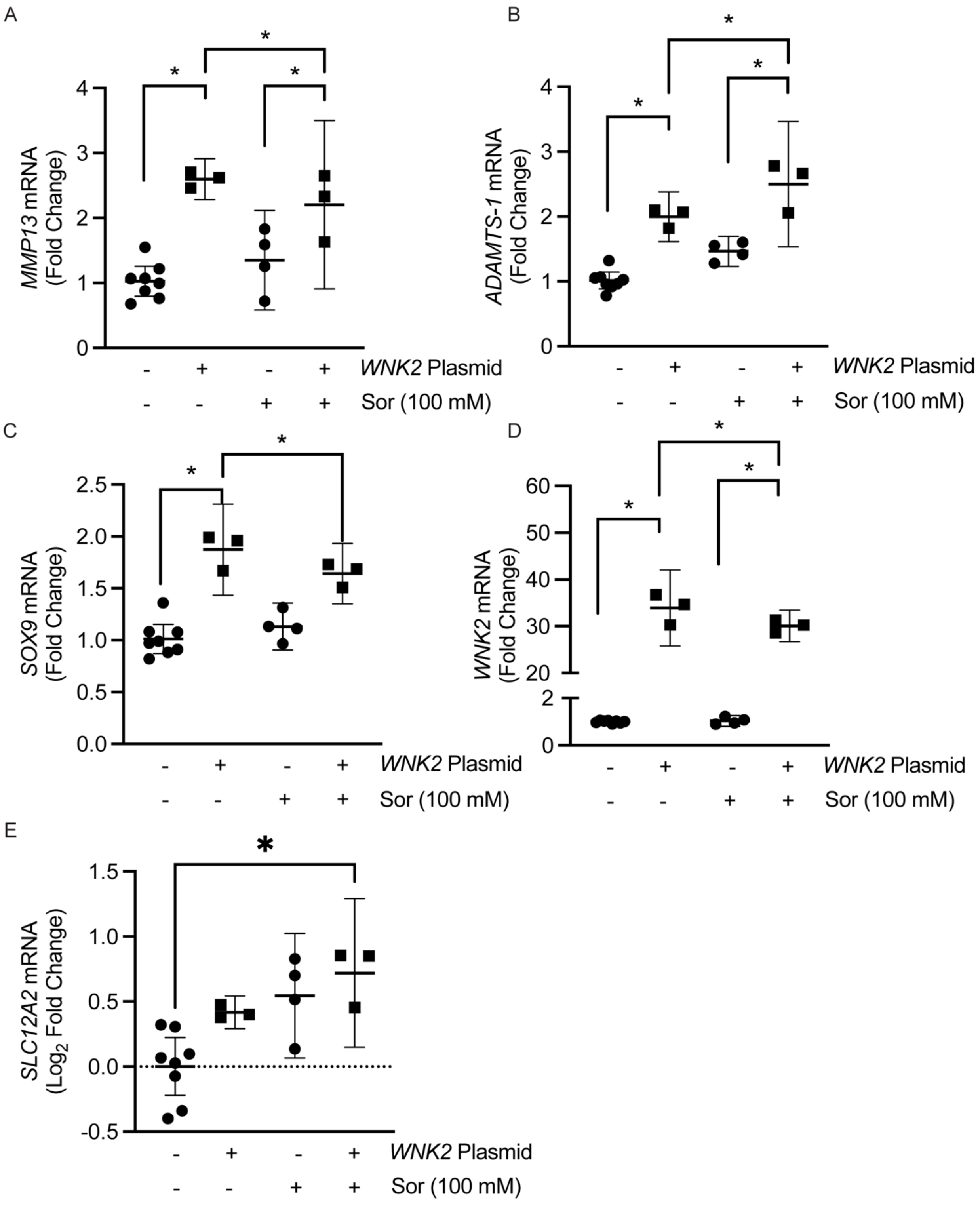

Supplementary Fig. 8 *WNK2* overexpression and hyperosmotic stress significantly alters the expression known OA-associated markers. T/C-28a2 human chondrocytes were transfected with *WNK2* for 48 hours and then treated for 7 days with 100 mM sorbitol (Sor). (A-C) qPCR analysis of OA-associated genes *MMP13, ADAMTS-1* and *SOX9*; (D) qPCR analysis of

WNK2 and (E) *SLC12A2*. Data are expressed as Fold Change (mean with 95% confidence interval) relative to control and untransfected T/C-28a2 human chondrocytes. n = 3-8 samples/condition. Error bars represent 95% confidence interval and a statistically significant difference of $p \leq 0.05$ (*) was determined by one-way ANOVA with Tukey's multiple comparisons test, except for *SLC12A2*, which a statistically significant difference of $p \leq 0.05$ (*) was determined by a Kruskal–Wallis test.

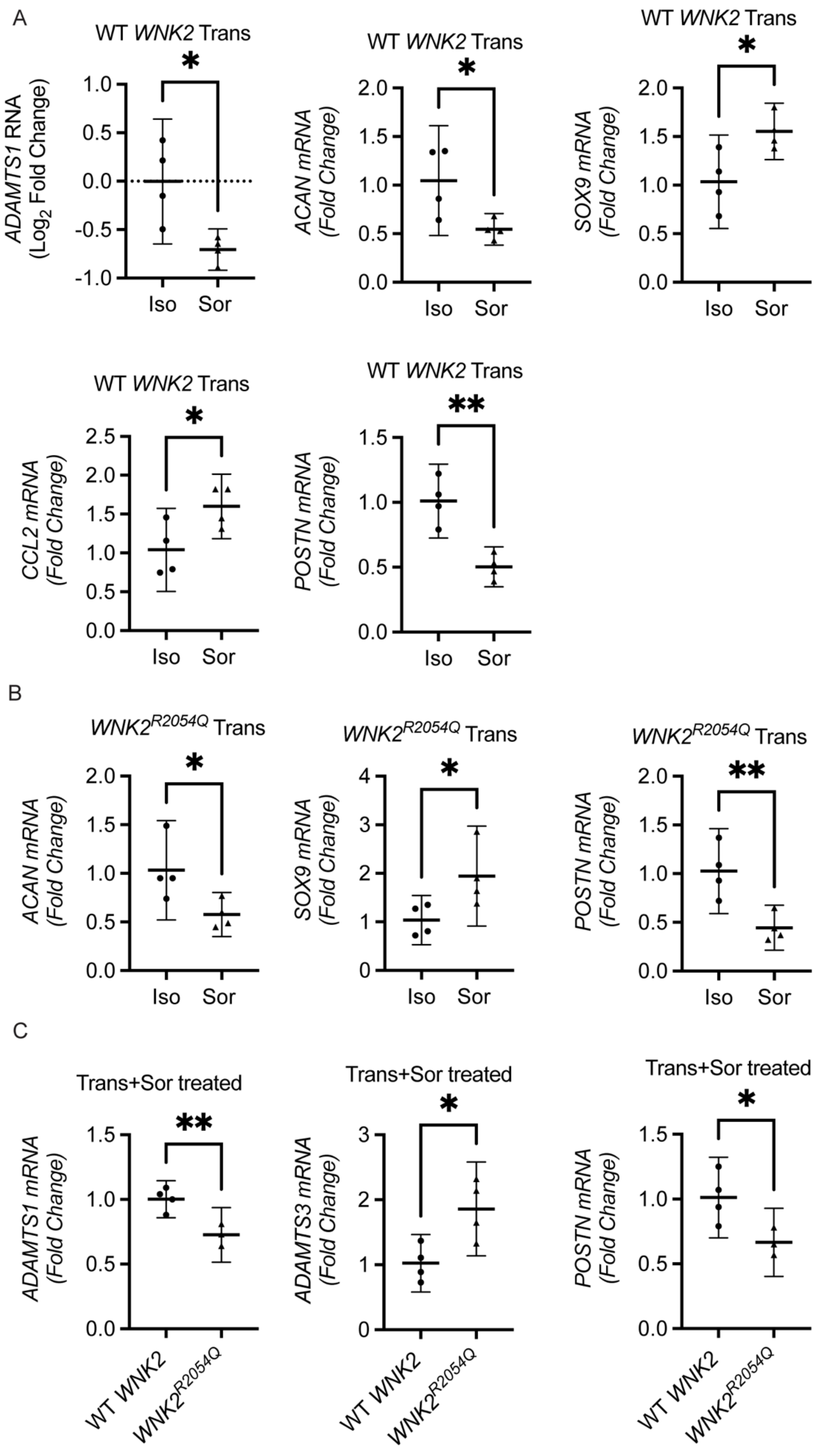
A
WT WNK2 Trans
*
ADAMTS1 RNA (Log2 Fold Change)
1.0
0.5
0.0
-0.5
-1.0
Iso
Sor
WT WNK2 Trans
*
ACAN mRNA (Fold Change)
2.0
1.5
1.0
0.5
0.0
Iso
Sor
WT WNK2 Trans
*
SOX9 mRNA (Fold Change)
2.0
1.5
1.0
0.5
0.0
Iso
Sor
WT WNK2 Trans
*
CCL2 mRNA (Fold Change)
2.5
2.0
1.5
1.0
0.5
0.0
Iso
Sor
WT WNK2 Trans
**
POSTN mRNA (Fold Change)
1.5
1.0
0.5
0.0
Iso
Sor
B
WNK2R2054Q Trans
*
ACAN mRNA (Fold Change)
2.0
1.5
1.0
0.5
0.0
Iso
Sor
WNK2R2054Q Trans
*
SOX9 mRNA (Fold Change)
4
3
2
1
0
Iso
Sor
WNK2R2054Q Trans
**
POSTN mRNA (Fold Change)
2.0
1.5
1.0
0.5
0.0
Iso
Sor
C
Trans+Sor treated
**
ADAMTS1 mRNA (Fold Change)
1.5
1.0
0.5
0.0
WT WNK2
WNK2R2054Q
Trans+Sor treated
*
ADAMTS3 mRNA (Fold Change)
3
2
1
0
WT WNK2
WNK2R2054Q
Trans+Sor treated
*
POSTN mRNA (Fold Change)
1.5
1.0
0.5
0.0
WT WNK2
WNK2R2054Q

Supplementary Fig. 9 Primary human chondrocytes have a similar transcriptional response to *WNK2* overexpression and hyperosmotic stress as compared to T/C-28a2 chondrocytes. (A-C) Primary human chondrocytes were electroporated with *WNK2* (WT or $WNK2^{R2054Q}$) and after 48 hours they were treated for 7 days with 100 mM sorbitol (Sor). qRT-PCR analysis was used to quantify gene expression. (A) Comparison of primary human chondrocytes overexpressing *WNK2* exposed to isotonic (Iso) or hyperosmotic (Sor) treatment for 7 days. (B) Comparison of primary human chondrocytes overexpressing $WNK2^{R2054Q}$ exposed to isotonic (Iso) or hyperosmotic (Sor) treatment for 7 days. (C) Comparison of primary human chondrocytes overexpressing WT or $WNK2^{R2054Q}$ and exposed to hyperosmotic (Sor) treatment for 7 days. (D) RNA-seq data form T/C-28a2 chondrocytes indicating expression of genes analyzed in A-C. NA indicates that *SOX9* mRNA was not detected in T/C-28a2 chondrocytes. Statistically significant differences of $p \leq 0.05$ (*) and $p \leq 0.01$ (**) were determined by a two-tailed unpaired t-test, n=4 biological replicates.

Ensembl	Gene	Ctrl *WNK2*$^{-}$ vs WT		Immediate response-2 hrs Sor				Late response-7 days Sor				Late response-7 days Sor			
				WT cells Sor treated vs Ctrl		Sor treated *WNK2*$^{-}$ vs WT		WT cells Sor treated vs Ctrl		Sor treated *WNK2*$^{-}$ vs WT		*WNK2*^{R2054Q} vs WT		Sor treated *WNK2*^{R2054Q} vs WT	
		Log2 FoldChange	padj	Log2 FoldChange	padj	Log2 FoldChange	padj	Log2 FoldChange	padj	Log2 FoldChange	padj	Log2 FoldChange	padj	Log2 FoldChange	padj
ENSG00000133794	ARNTL	0.262	0.063	0.727	0.000	-0.200	0.029	0.397	0.018	-0.046	0.793	0.044	0.830	-0.350	0.003
ENSG00000134852	CLOCK	1.004	0.001	0.234	0.068	0.156	0.283	0.629	0.140	0.486	0.158	0.435	0.276	-1.101	0.000
ENSG00000170485	NPAS2	0.344	0.016	-0.883	0.000	1.422	0.000	0.844	0.000	0.987	0.000	0.143	0.563	-0.644	0.000
ENSG00000179094	PER1	-0.397	0.069	-0.312	0.001	-0.167	0.152	0.411	0.135	-1.138	0.000	-0.342	0.300	0.710	0.004
ENSG00000132326	PER2	0.587	0.077	0.174	0.504	0.219	0.412	0.275	0.620	0.127	0.761	0.196	0.680	-0.261	0.489
ENSG00000049246	PER3	0.046	0.884	-0.267	0.018	-0.194	0.160	0.359	0.241	-0.513	0.018	0.212	0.470	-0.598	0.006
ENSG00000008405	CRY1	0.450	0.000	0.757	0.000	0.095	0.179	-0.020	0.919	0.524	0.000	0.049	0.801	-0.131	0.334
ENSG00000126368	NR1D1	-0.448	0.007	0.374	0.000	-0.205	0.100	0.312	0.152	-0.547	0.000	-0.464	0.008	0.323	0.036
ENSG00000174738	NR1D2	-0.588	0.000	0.690	0.000	-0.598	0.000	-0.059	0.855	-0.556	0.001	0.139	0.553	-0.353	0.031
ENSG00000069667	RORA	0.633	0.047	-0.674	0.008	0.111	0.763	0.590	0.153	-0.037	0.932	0.545	0.172	-1.209	0.000
ENSG00000143365	RORC	-0.424	0.892	0.828	0.740	1.504	0.463	0.368	NA	0.544	0.845	0.441	NA	-1.196	NA
ENSG00000105516	DBP	-1.059	0.004	-0.273	0.288	-0.554	0.041	0.128	0.856	-1.545	0.000	-0.757	0.085	1.018	0.005
ENSG00000159208	CIART	-0.033	0.941	-1.299	0.000	0.549	0.050	0.305	0.546	-0.480	0.129	0.100	0.822	0.886	0.002
ENSG00000165030	NFIL3	-0.198	0.227	1.105	0.000	-0.374	0.000	-0.011	0.973	-0.077	0.682	-0.222	0.280	0.030	0.892
ENSG00000167074	TEF	-0.702	0.000	-0.305	0.011	0.031	0.866	0.110	0.679	-1.056	0.000	-0.341	0.187	0.698	0.001
ENSG00000108924	HLF	2.823	0.201	4.701	0.000	0.139	0.899	2.231	NA	2.021	0.302	1.034	NA	1.017	NA
ENSG00000036672	USP2	-1.505	0.007	-0.763	0.160	-0.906	0.188	-0.729	0.370	-0.541	0.412	0.043	0.970	1.371	0.066

Supplementary Table 7. Expression of 17 central circadian clock genes in response to acute (2 hour sorbitol treatment) and chronic (7-day sorbitol treatment) hyperosmotic stress in our RNA-seq datasets from WT, *WNK2*$^{-}$, and *WNK2*^{R2054Q} overexpressing cells T/C-28a2 chondrocytes.

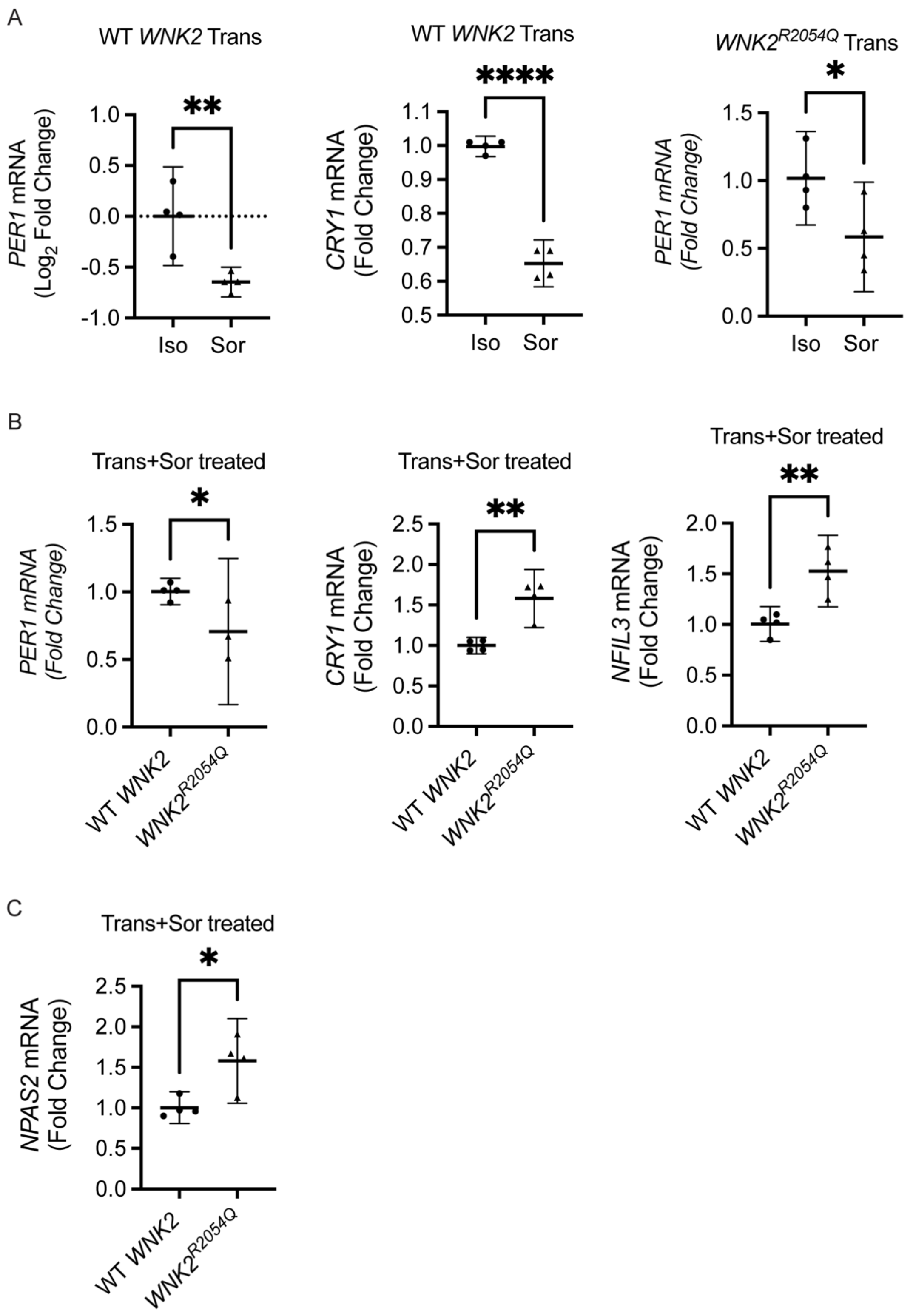
A
WT WNK2 Trans
PER1 mRNA (Log2 Fold Change)
**
Iso
Sor
WT WNK2 Trans
CRY1 mRNA (Fold Change)
****
WNK2R2054Q Trans
PER1 mRNA (Fold Change)
*
B
Trans+Sor treated
PER1 mRNA (Fold Change)
CRY1 mRNA (Fold Change)
NFIL3 mRNA (Fold Change)
WT WNK2
WNK2R2054Q
C
Trans+Sor treated
NPAS2 mRNA (Fold Change)
*

Supplementary Figure 10. Expression of circadian clock genes in primary human chondrocytes in response to *WNK2* overexpression and hyperosmotic stress. (A-B) Primary human chondrocytes were electroporated with *WNK2* (WT or $WNK2^{R2054Q}$) and after 48 hours they were treated for 7 days with 100 mM sorbitol (Sor). qRT-PCR analysis was used to quantify gene expression. (A) Comparison of primary human chondrocytes overexpressing *WNK2* exposed to isotonic (Iso) or hyperosmotic (Sor) treatment for 7 days. (B) Comparison of primary human chondrocytes overexpressing WT or $WNK2^{R2054Q}$ and exposed to hyperosmotic (Sor) treatment for 7 days. Statistically significant differences of $p \leq 0.05$ (*), $p \leq 0.01$ (**), and $p \leq 0.001$ (***) were determined by a two-tailed unpaired t-test, n=4 biological replicates.

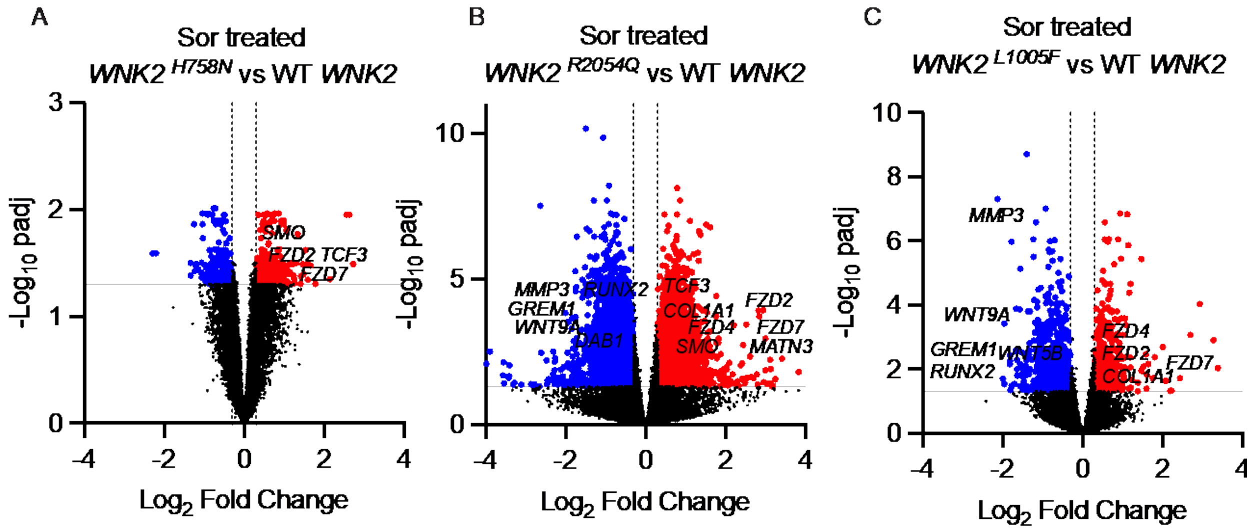

Supplementary Fig. 11 OA-associated *WNK2* variants amply the chondrocyte response to hyperosmotic stress. (A-C) The volcano plots indicate genes significantly upregulated (red) or downregulated (blue) in WT chondrocytes expressing $WNK2^{H758N}$, $WNK2^{R2054Q}$, or $WNK2^{L1005F}$ compared with WT *WNK2* expressing chondrocytes.

REFERENCES

1. Gavile, C.M., Kazmers, N.H., Novak, K.A., Meeks, H.D., Yu, Z., Thomas, J.L., Hansen, C., Barker, T., and Jurynec, M.J. (2022). Familial Clustering and Genetic Analysis of Severe Thumb Carpometacarpal Joint Osteoarthritis in a Large Statewide Cohort. J Hand Surg Am *47*, 923-933. 10.1016/j.jhsa.2022.08.004.
2. Jurynec, M.J., Gavile, C.M., Honeggar, M., Ma, Y., Veerabhadraiah, S.R., Novak, K.A., Hoshijima, K., Kazmers, N.H., and Grunwald, D.J. (2022). NOD/RIPK2 signalling pathway contributes to osteoarthritis susceptibility. Ann Rheum Dis *81*, 1465-1473. 10.1136/annrheumdis-2022-222497.
3. Kazmers, N.H., Meeks, H.D., Novak, K.A., Yu, Z., Fulde, G.L., Thomas, J.L., Barker, T., and Jurynec, M.J. (2021). Familial Clustering of Erosive Hand Osteoarthritis in a Large Statewide Cohort. Arthritis Rheumatol *73*, 440-447. 10.1002/art.41520.
4. Jurynec, M.J., Nosyreva, E., Thompson, D., Munoz, C., Novak, K.A., Matheson, D.J., Kazmers, N.H., and Syeda, R. (2024). PIEZO1 variants that reduce open channel probability are associated with familial osteoarthritis. medRxiv. 10.1101/2024.09.03.24312969.
5. Kazmers, N.H., Yu, Z., Barker, T., Abraham, T., Romero, R., and Jurynec, M.J. (2020). Evaluation for Kienbock Disease Familial Clustering: A Population-Based Cohort Study. J Hand Surg Am *45*, 1-8 e1. 10.1016/j.jhsa.2019.10.005.
6. Jurynec, M.J., Sawitzke, A.D., Beals, T.C., Redd, M.J., Stevens, J., Otterud, B., Leppert, M.F., and Grunwald, D.J. (2018). A hyperactivating proinflammatory RIPK2 allele associated with early-onset osteoarthritis. Hum Mol Genet *27*, 2406. 10.1093/hmg/ddy196.
7. Wang, K., Li, M., and Hakonarson, H. (2010). ANNOVAR: functional annotation of genetic variants from high-throughput sequencing data. Nucleic Acids Res *38*, e164. 10.1093/nar/gkq603.
8. Hu, H., Roach, J.C., Coon, H., Guthery, S.L., Voelkerding, K.V., Margraf, R.L., Durtschi, J.D., Tavtigian, S.V., Shankaracharya, Wu, W., et al. (2014). A unified test of linkage analysis and rare-variant association for analysis of pedigree sequence data. Nat Biotechnol *32*, 663-669. 10.1038/nbt.2895.
9. Singleton, M.V., Guthery, S.L., Voelkerding, K.V., Chen, K., Kennedy, B., Margraf, R.L., Durtschi, J., Eilbeck, K., Reese, M.G., Jorde, L.B., et al. (2014). Phevor combines multiple biomedical ontologies for accurate identification of disease-causing alleles in single individuals and small nuclear families. Am J Hum Genet *94*, 599-610. 10.1016/j.ajhg.2014.03.010.
10. Maza, E., Frasse, P., Senin, P., Bouzayen, M., and Zouine, M. (2013). Comparison of normalization methods for differential gene expression analysis in RNA-Seq experiments: A matter of relative size of studied transcriptomes. Commun Integr Biol *6*, e25849. 10.4161/cib.25849.
11. Love, M.I., Huber, W., and Anders, S. (2014). Moderated estimation of fold change and dispersion for RNA-seq data with DESeq2. Genome Biol *15*, 550. 10.1186/s13059-014-0550-8.
12. Boyd-Shiwarski, C.R., Shiwarski, D.J., Griffiths, S.E., Beacham, R.T., Norrell, L., Morrison, D.E., Wang, J., Mann, J., Tennant, W., Anderson, E.N., et al. (2022). WNK

kinases sense molecular crowding and rescue cell volume via phase separation. Cell *185*, 4488-4506 e4420. 10.1016/j.cell.2022.09.042.
13. Yamada, K., Park, H.M., Rigel, D.F., DiPetrillo, K., Whalen, E.J., Anisowicz, A., Beil, M., Berstler, J., Brocklehurst, C.E., Burdick, D.A., et al. (2016). Small-molecule WNK inhibition regulates cardiovascular and renal function. Nat Chem Biol *12*, 896-898. 10.1038/nchembio.2168.
14. Christiansen, B.A., Guilak, F., Lockwood, K.A., Olson, S.A., Pitsillides, A.A., Sandell, L.J., Silva, M.J., van der Meulen, M.C., and Haudenschild, D.R. (2015). Non-invasive mouse models of post-traumatic osteoarthritis. Osteoarthritis Cartilage *23*, 1627-1638. 10.1016/j.joca.2015.05.009.
15. Piala, A.T., Moon, T.M., Akella, R., He, H., Cobb, M.H., and Goldsmith, E.J. (2014). Chloride sensing by WNK1 involves inhibition of autophosphorylation. Sci Signal *7*, ra41. 10.1126/scisignal.2005050.
16. Livak, K.J., and Schmittgen, T.D. (2001). Analysis of relative gene expression data using real-time quantitative PCR and the 2(-Delta Delta C(T)) Method. Methods *25*, 402-408. 10.1006/meth.2001.1262.
17. Anders, S., Pyl, P.T., and Huber, W. (2015). HTSeq--a Python framework to work with high-throughput sequencing data. Bioinformatics *31*, 166-169. 10.1093/bioinformatics/btu638.
18. Fisch, K.M., Gamini, R., Alvarez-Garcia, O., Akagi, R., Saito, M., Muramatsu, Y., Sasho, T., Koziol, J.A., Su, A.I., and Lotz, M.K. (2018). Identification of transcription factors responsible for dysregulated networks in human osteoarthritis cartilage by global gene expression analysis. Osteoarthritis Cartilage *26*, 1531-1538. 10.1016/j.joca.2018.07.012.
19. Ramos, Y.F., den Hollander, W., Bovee, J.V., Bomer, N., van der Breggen, R., Lakenberg, N., Keurentjes, J.C., Goeman, J.J., Slagboom, P.E., Nelissen, R.G., et al. (2014). Genes involved in the osteoarthritis process identified through genome wide expression analysis in articular cartilage; the RAAK study. PLoS One *9*, e103056. 10.1371/journal.pone.0103056.
20. Reed, K.S.M., Ulici, V., Kim, C., Chubinskaya, S., Loeser, R.F., and Phanstiel, D.H. (2021). Transcriptional response of human articular chondrocytes treated with fibronectin fragments: an in vitro model of the osteoarthritis phenotype. Osteoarthritis Cartilage *29*, 235-247. 10.1016/j.joca.2020.09.006.
21. Steinberg, J., Ritchie, G.R.S., Roumeliotis, T.I., Jayasuriya, R.L., Clark, M.J., Brooks, R.A., Binch, A.L.A., Shah, K.M., Coyle, R., Pardo, M., et al. (2017). Integrative epigenomics, transcriptomics and proteomics of patient chondrocytes reveal genes and pathways involved in osteoarthritis. Sci Rep *7*, 8935. 10.1038/s41598-017-09335-6.
22. Wang, K., Esbensen, Q.Y., Karlsen, T.A., Eftang, C.N., Owesen, C., Aroen, A., and Jakobsen, R.B. (2021). Low-Input RNA-Sequencing in Patients with Cartilage Lesions, Osteoarthritis, and Healthy Cartilage. Cartilage *13*, 5505-5625.
23. Chou, C.H., Wu, C.C., Song, I.W., Chuang, H.P., Lu, L.S., Chang, J.H., Kuo, S.Y., Lee, C.H., Wu, J.Y., Chen, Y.T., et al. (2013). Genome-wide expression profiles of subchondral bone in osteoarthritis. Arthritis Res Ther *15*, R190. 10.1186/ar4380.
24. Wollbold, J., Huber, R., Pohlers, D., Koczan, D., Guthke, R., Kinne, R.W., and Gausmann, U. (2009). Adapted Boolean network models for extracellular matrix formation. BMC Syst Biol *3*, 77. 10.1186/1752-0509-3-77.

25. Karouzakis, E., Trenkmann, M., Gay, R.E., Michel, B.A., Gay, S., and Neidhart, M. (2014). Epigenome analysis reveals TBX5 as a novel transcription factor involved in the activation of rheumatoid arthritis synovial fibroblasts. J Immunol *193*, 4945-4951. 10.4049/jimmunol.1400066.
26. Soul, J., Hardingham, T.E., Boot-Handford, R.P., and Schwartz, J.M. (2019). SkeletalVis: an exploration and meta-analysis data portal of cross-species skeletal transcriptomics data. Bioinformatics *35*, 2283-2290. 10.1093/bioinformatics/bty947.
